# Prevalence, Incidence, and Risk of Different Comorbidity Categories in Pediatric Multiple Sclerosis: A Systematic Review and Meta-Analysis Protocol

**DOI:** 10.3390/children13020307

**Published:** 2026-02-23

**Authors:** Sara Samadzadeh, Moein Mirzai, Aysan Valinejad Qanati, Andrea Icks, Charalabos-Markos Dintsios

**Affiliations:** 1 Department of Neurology, University Hospital Düsseldorf, Medical Faculty, Heinrich Heine University Düsseldorf, Moorenstrasse 5, 40225 Düsseldorf, Germany; 2 Experimental and Clinical Research Center (ECRC), a Cooperation Between Charité–Universitätsmedizin Berlin and the Max Delbrück Center for Molecular Medicine in the Helmholtz Association (MDC), Charité–Universitätsmedizin Berlin, Corporate Member of Freie Universität Berlin and Humboldt-Universität zu Berlin, Lindenberger Weg 80, 13125 Berlin, Germany; 3 School of Medicine, Tehran University of Medical Sciences, Tehran 1416753955, Iran; 4 Institute for Health Services Research and Health Economics, Centre for Health and Society, Medical Faculty, Heinrich Heine University, Building: 17.11, Moorenstr. 5, 40225 Düsseldorf, Germany; 5 Institute for Health Services Research and Health Economics, German Diabetes Center, Leibniz Center for Diabetes Research at Heinrich Heine University Düsseldorf, Düsseldorf, Auf’m Hennekamp 65, 40225 Düsseldorf, Germany; 6 German Center for Diabetes Research (DZD), Partner Düsseldorf, Ingolstaedter Landstraße 1, 85764 Neuherberg, Germany

**Keywords:** pediatric-onset multiple sclerosis (POMS), pediatric MS, comorbidities, prevalence, incidence, risk, systematic review, meta-analysis, disease progression, clinical outcomes

## Abstract

**Highlights:**

**What are the main features of this protocol?**

- This protocol outlines a systematic and reproducible approach to identify evidence on comorbidities in pediatric-onset multiple sclerosis (POMS).
- A comprehensive search strategy covering 15 comorbidity categories, predefined eligibility criteria, and independent screening and extraction is specified.

**What are the implications of this protocol?**

- The protocol ensures methodological transparency and replicability, facilitating robust synthesis and future updates.
- It provides a standardized framework for quantifying comorbidity prevalence, incidence, and risk in POMS to inform future clinical and research efforts.

**Abstract:**

Background/Objectives: Pediatric-onset multiple sclerosis (POMS), defined as onset before age 18, is increasingly recognized as a distinct entity, often associated with a more burdensome disease course and earlier disability milestones than adult-onset MS. Although comorbidities may significantly affect disease progression and outcomes, their prevalence, incidence, risk, and characteristics in POMS remain poorly understood. To date, no systematic review has comprehensively evaluated comorbidities in POMS. The primary aim is to systematically identify and synthesize available evidence on the prevalence, incidence, risk, and characteristics of these comorbidities in POMS populations, as well as any reported effects on disease course, treatment outcomes, and overall clinical management. Methods: We will conduct a systematic review and meta-analysis following a hierarchical and pragmatic analytical strategy tailored to the expected heterogeneity and limited evidence base in POMS. MEDLINE (via PubMed) and Embase (produced by Elsevier) will be searched without date restrictions, combining controlled vocabulary terms (MeSH/Emtree) and relevant keywords for POMS and 15 predefined comorbidity categories. Study selection, abstract and full-text screening, and data extraction will be performed independently by two reviewers using predefined criteria and standardized forms. The primary quantitative outcome will be the pooled prevalence of comorbidities. Where study design and reporting permit, incidence rates will be assessed as secondary outcomes, and risk estimates (e.g., odds ratios) will be evaluated only in studies with appropriate comparator groups. Meta-analyses will be conducted using random-effects models when pooling is feasible. Heterogeneity will be assessed using the I^2^ statistic and Cochran’s Q test, with sensitivity and subgroup analyses performed only when sufficient data are available. When quantitative synthesis is not appropriate due to limited data or substantial heterogeneity, findings will be summarized descriptively. Publication bias will be evaluated using funnel plots and, where applicable, Egger’s and Begg’s tests. This protocol adheres to PRISMA and PRISMA-P guidelines. Discussion: A systematic quantification of comorbidity prevalence, incidence (where available), and risk, together with POMS-specific characteristics and any reported impact on clinical outcomes, is anticipated to provide a crucial evidence base for guiding screening, refining management strategies, and informing future research directions. Ultimately, these findings may improve clinical outcomes and quality of life for children and adolescents with MS.

## 1. Introduction

Multiple sclerosis (MS) is a chronic autoimmune inflammatory demyelinating disease of the central nervous system, predominantly affecting adults. However, pediatric-onset MS (POMS), defined as disease onset before the age of 18, is increasingly recognized as a distinct entity, accounting for approximately 2% to 10% of all MS cases [1,2]. Although POMS is less common than adult-onset MS (AOMS), it has distinct clinical features and challenges that necessitate specialized diagnostic and treatment approaches.

Pediatric MS typically begins around age 12, with 17–30% of cases occurring before age 10. Pre-pubescent MS is rare. It affects boys and girls equally before puberty but shifts post-puberty, with a rising female predominance, making MS significantly more common in girls during adolescence [3,4]. Epidemiological studies indicate that the global prevalence of POMS ranges from 0.69 to 26.92 per 100,000 individuals, while its incidence varies between 0.05 and 2.85 per 100,000 individuals annually [5].

Beyond its prevalence, POMS is characterized by a more aggressive disease course than AOMS, with a high relapse rate, rapid white and gray matter damage, and early disability accumulation, leading to significant physical and cognitive impairment [6]. It commonly presents with focal neurological deficits, transverse myelitis, ataxia, optic neuritis, brainstem symptoms, and acute demyelinating encephalomyelitis (ADEM) [7]. Nearly all cases follow a relapsing-remitting course, and signs of progression should prompt evaluation for alternative diagnoses [3]. Due to its early onset and impact on neurological development, POMS significantly affects quality of life (QoL), leading to challenges in school performance, social interactions, and physical activities [8,9].

Meanwhile, although POMS-specific diagnostic criteria are outdated [10], the 2017 McDonald criteria for AOMS [11,12] are widely used, enhancing early diagnosis and treatment initiation [3,13]. Nevertheless, despite its severity and importance, POMS lacks updated treatment guidelines, leading to significant variability in therapeutic approaches [6,14,15]. Older recommendations favored starting with interferon-beta or glatiramer acetate before escalating to stronger therapies [16,17,18,19]. However, accumulating evidence from recent studies suggests that newer high-efficacy disease-modifying treatments (DMTs), such as natalizumab, fingolimod, rituximab, alemtuzumab, and ocrelizumab, are more effective in reducing relapses, disability progression, and MRI activity compared to moderate-efficacy DMTs [3,14,20,21]. With growing data supporting their superior clinical outcomes, better tolerability, and lower discontinuation rates, there is a shift toward prioritizing early use of high-efficacy DMTs rather than following a stepwise escalation approach [22,23]. While current safety data are reassuring, long-term studies are needed for further evaluation.

In addition, diagnosing and managing POMS remains particularly complex due to several factors. The disease overlaps with other neuroinflammatory disorders [24] and has distinct pathophysiology compared to AOMS [22,25,26,27]. Furthermore, comorbid conditions [28], including psychiatric disorders (e.g., depression, anxiety) [29,30,31,32], autoimmune diseases (e.g., type 1 diabetes, autoimmune thyroid disease) [33,34,35], and metabolic syndromes (e.g., childhood obesity) [36], further complicate disease progression, therapeutic decisions, overall patient outcomes and quality of life [37]. These conditions may exacerbate neuroinflammation, complicate immunological pathways, and diminish the effectiveness of DMTs, ultimately increasing the risk of early disability. Moreover, diagnosing comorbidities in POMS is challenging, as overlapping symptoms may blur the line between MS activity and separate pathological processes. Despite the recognized importance of comorbidities in POMS, there is currently no existing systematic review focusing on their prevalence, incidence, and risk in this population. A comprehensive evaluation of available data is therefore needed to establish a sound evidence base for clinical management and inform future research. The aim of this systematic review and meta-analysis is to synthesize and critically appraise the existing literature on comorbidities in pediatric MS, estimating their prevalence, incidence, and risk, while clarifying their impact on disease course, treatment outcomes, and quality of life.

## 2. Materials and Methods

### 2.1. Protocol

This protocol follows the guidelines outlined in the Preferred Reporting Items for Systematic Reviews and Meta-Analyses (PRISMA) [38] and the PRISMA for systematic review protocols (PRISMA-P) [39]. Additionally, this protocol has been registered with the Open Science Framework (OSF) (https://doi.org/10.17605/OSF.IO/QU7X8). Based on the Population, Exposure, Outcome (PEO) framework, our research question is: In children and adolescents with multiple sclerosis (POMS; pediatric-onset multiple sclerosis), what are the prevalence, incidence, and risk associated with 15 specified comorbid conditions, and how do these comorbidities affect the disease trajectory, treatment efficacy, and clinical management?

### 2.2. Study Outcomes

Primary:

The outcome of interest will be the prevalence, incidence, or risk of 15 different categories of comorbid conditions in pediatric patients with multiple sclerosis.

Secondary (Stratification by Demographics and Study Factors):

We will evaluate the following secondary outcomes if the data are available:

1. Prevalence, incidence, and risk of the specified comorbid conditions based on the age of pediatric MS patients.
2. Prevalence, incidence, and risk of the specified comorbid conditions before and after MS onset in pediatric patients.
3. Prevalence, incidence, and risk of the specified comorbid conditions based on the sex of pediatric MS patients.
4. Prevalence, incidence, and risk of the specified comorbid conditions based on the year of publication.
5. Prevalence, incidence, and risk of the specified comorbid conditions in relation to Disease-Modifying Therapies used in pediatric MS.
6. Prevalence, incidence, and risk of the specified comorbid conditions based on the patients’ source (population-based/non-population-based studies).
7. Prevalence, incidence, and risk of the specified comorbid conditions based on study design (Cohort, case–control, cross-sectional).
8. Prevalence, incidence, and risk of the specified comorbid conditions based on the study region (North America, South America, Europe, Asia, other).
9. Prevalence, incidence, and risk of the specified comorbid conditions based on the quality assessment of included studies.
10. Identifying the source of possible heterogeneity among study findings.

Complementary:

When sufficient data are available, the clinical and demographic characteristics of comorbidities in pediatric MS will be described, and their potential impact on disease trajectory, treatment decisions, and quality of life will be assessed. If pooling is unfeasible, a narrative synthesis will be conducted to highlight key insights into the interplay between comorbidities and pediatric MS outcomes.

### 2.3. Comorbidity Categories

The following table (Table 1) lists the primary categories of comorbid conditions of interest for this review, along with any additional relevant conditions identified in the literature.

### 2.4. Eligibility Criteria

The systematic review will encompass all studies that examine the primary outcomes of interest, which are the prevalence, incidence, or risk of 15 different categories of comorbid conditions in pediatric patients with multiple sclerosis. Detailed inclusion criteria are provided in Table 2.

### 2.5. Data Sources

Our search strategy will focus on leveraging key databases to pinpoint relevant studies. Specifically, we will conduct systematic searches in PubMed (provided by the National Library of Medicine) and Embase (produced by Elsevier).

### 2.6. Search Strategy and Feasibility Assessment

This review will be conducted using searches in two mentioned data sources without time restrictions, employing a combination of search strategies, Boolean operators, and both controlled vocabulary and keywords provided by the databases. We have developed our “primary” bibliographic search strategy in accordance with PubMed search rules, which will be adapted for use in other database formats [40]. The search was organized around 18 domains of keywords and Medical Subject Headings (MeSH), with two main domains focusing on pediatric multiple sclerosis and the remainder addressing different comorbidity categorizations. Additionally, MeSH terms will not be used when there is an overlap between pediatric MS symptoms or categories, such as neurological diseases or immunological disorders. Furthermore, the primary search will prioritize full disease names over abbreviations, with a supplementary sensitivity search for selected key acronyms (e.g., ADHD, T1D/T2D, IBD). Details of this search strategy can be found in Supplementary Tables S1 and S2.

### 2.7. Technical Tools

The outcomes of the systematic literature search will be compiled and managed using EndNote. Subsequently, these references will be transferred from the bibliographic database into Covidence, a widely used software tool designed to streamline the processes of screening and data extraction for systematic reviews. Covidence allows for efficient management of literature review stages from the initial import of citations to the final data synthesis. It will enable multiple reviewers to independently extract data from the included studies. During the data extraction phase, we will utilize customized spreadsheet forms tailored to our specific data needs. This approach will facilitate the accurate and systematic collection of pertinent data from each study. All gathered information will be shared and made accessible to the research team, which is composed of two pairs of researchers working in tandem. 

### 2.8. Study Selection

Two independent investigators will execute the searches across databases, consolidating the outcomes and eliminating redundancies. Subsequently, two independent reviewers will undertake a preliminary screening of the titles and abstracts against predetermined inclusion and exclusion criteria. Articles deemed potentially relevant will undergo a comprehensive full-text review by these reviewers to validate adherence to the criteria. In instances of divergent assessments between reviewers, deliberations will be held to achieve a consensus on study eligibility.

### 2.9. Additional Data Source Selection

During the screening of titles and abstracts, we will identify systematic literature reviews that focus on pediatric multiple sclerosis, considering them as potential sources of interest. Subsequent to the full-text review, we will compile the reference lists from all identified publications for data extraction purposes. Additionally, we will track forward citations of these publications using various citation databases. We will also utilize PubMed’s “related articles” feature to gather the first 10 related articles for each paper. This limitation on the number of articles is intentionally set to minimize the review burden. In addition, author-based searches of recognized experts in pediatric MS and comorbidity research will be performed to identify potentially relevant publications not captured through database queries alone. All references obtained in this phase will undergo the same selection procedures as outlined in the “Study Selection” section.

### 2.10. Data Extraction

Two investigators will independently undertake data extraction from the selected studies. In the event of discrepancies between their evaluations, deliberative discussions will be held to attain a unified consensus. Data parameters for extraction from each eligible study are delineated in Table 3.

### 2.11. Quality Appraisal

For this systematic review, the quality appraisal will be rigorously conducted to ensure the reliability and validity of the included studies. Essential components will encompass a clear definition of the research question, systematic and comprehensive database searches, consistent data extraction by two independent reviewers, and an assessment of risk of bias using validated tools. The synthesis of results will consider heterogeneity and potential publication bias, while conclusions will align with the evidence presented. Throughout the process, transparency will be maintained, including adherence to PRISMA guidelines, provision of a flow diagram, and clear declarations of any conflicts of interest.

Two reviewers will independently appraise the methodological quality of the included studies in the systematic review on pediatric MS utilizing the Joanna Briggs Institute (JBI) critical appraisal tool [41]. Funnel plots, which plot effect estimates against their standard errors, will serve as the primary visual tool for detecting potential asymmetry suggestive of publication bias. For a more quantitative assessment, both Begg’s rank correlation test and Egger’s linear regression test will be employed. A statistically significant *p*-value (less than 0.05) from these tests would suggest potential publication bias.

### 2.12. Assessment of Heterogeneity

To assess the heterogeneity across the studies included for primary analysis, several statistical tools and visual methods will be utilized. Firstly, forest plots will be visually inspected to provide a graphical representation of the individual study estimates in relation to the overall pooled estimate. Quantitatively, the I2 statistic will be employed, which denotes the proportion of the total variation attributable to between-study heterogeneity, rather than random chance. Interpretatively, I2 values of 25%, 50%, and 75% signify low, moderate, and high heterogeneity, respectively. Alongside this, Cochran’s Q statistic will be calculated, with a *p*-value less than 0.10 indicating statistically significant heterogeneity. In instances where the I2 value exceeds 50%, suggesting considerable heterogeneity, a sensitivity analysis will be conducted. This involves sequentially removing individual studies to ascertain potential sources of this heterogeneity. Such variations can arise due to discrepancies in study design, statistical methods employed, participant characteristics, or other intrinsic factors.

### 2.13. Data Synthesis and Statistical Analysis

Analyses will follow a hierarchical and pragmatic strategy reflecting the expected heterogeneity and limited evidence base in pediatric-onset multiple sclerosis. The primary quantitative objective will be the estimation of the pooled prevalence of comorbidities. Where study design and reporting permit, incidence will be evaluated as a secondary outcome, and risk estimates (e.g., odds ratios) will be assessed only in studies with appropriate comparator groups. If data are insufficient for specific quantitative analyses, synthesis will first proceed descriptively. Priority will be given to the assessment of overall comorbidity burden (any comorbidity; single vs. multiple/comorbidity count) and major comorbidity categories before attempting analyses of individual conditions. Subgroup analyses will be undertaken only when an adequate number of studies per stratum are available; otherwise, findings will be integrated within broader categories and reported narratively.

The following analytical steps will be implemented in accordance with this hierarchy. We will begin by providing a detailed descriptive overview of each included study, documenting its design, sample size, population characteristics, observed comorbidities, and primary outcomes. When appropriate and warranted by study homogeneity, meta-analytic techniques will be employed to generate pooled estimates. The pooled prevalence of comorbidities in POMS will constitute the primary quantitative outcome, followed, where data permit, by incidence and subsequently by risk estimates, in line with the hierarchical strategy adopted for this review.

Pooled prevalence and incidence rates will be calculated, along with corresponding 95% confidence intervals (CIs). Incidence rates will be expressed as the number of new cases per person-years at risk and pooled using a random-effects model. Risk estimates (OR, RR, or HR) will be extracted only from studies that include a clearly defined comparator group. Acceptable comparators will include: (1) population-based or age- and sex-matched controls, (2) healthy siblings or community controls, and (3) disease controls when clearly justified (e.g., other pediatric non-MS neurological conditions). Comparisons with adult-onset MS will not be used as comparators. The effect measure will be analyzed as reported, and ORs, RRs, and HRs will not be treated as interchangeable; pooling will be performed only among studies using the same effect measure and comparable comparator groups. The choice between fixed- or random-effects models will be informed by the degree of heterogeneity, which will be evaluated via Cochran’s Q test and the I^2^ statistic. In cases of substantial heterogeneity (I^2^ > 50%), random-effects models will be preferred, and sensitivity analyses will be performed—such as excluding certain study types or measurement scales—to explore potential sources of variability. When the number of included studies is small (e.g., fewer than five) or when marked clinical or methodological heterogeneity is present, quantitative pooling will not be undertaken and a narrative synthesis will be performed instead. Studies reporting zero cases will not be excluded; such data will be addressed using approaches appropriate for rare events, with sensitivity analyses to assess the influence of analytic choices, and where zero-event data are too sparse or heterogeneous, findings will be summarized narratively. Outliers will be evaluated based on their effect direction and weight in the model, and sensitivity analyses will be performed to assess their influence. Additional sensitivity analyses may include excluding specific study types or measurement scales. Subgroup analyses may also be performed, contingent on data availability, to explore differences according to demographic factors, clinical manifestations, geographic distribution, and specific comorbidity categories, while avoiding underpowered comparisons.

If ten or more studies are included in any given meta-analysis, publication bias will be evaluated both visually and quantitatively. Funnel plots (plotting effect estimates against their standard errors) will serve as the primary visual tool for identifying asymmetry suggestive of publication bias. Begg’s rank correlation test and Egger’s linear regression test will provide quantitative assessments, with a *p*-value < 0.05 considered indicative of bias. When data are insufficiently homogeneous or cannot be pooled, we will present a narrative synthesis that highlights the range, frequency, and implications of identified comorbidities in POMS, emphasizing notable patterns, inconsistencies, and knowledge gaps.

All statistical analyses will be carried out using R software (version ≥ 4.0.0; including the meta, metafor, tidyverse, and ggplot2 packages) and/or Review Manager (RevMan, version 5.4 or later) for meta-analytic procedures, data management, and visualization.

### 2.14. Interpretation Considerations

For comorbidities potentially influenced by treatment (e.g., hypertension, diabetes, dyslipidemia), prevalence estimates will be interpreted with attention to possible iatrogenic contributions, particularly exposure to corticosteroids or DMTs, which may induce metabolic or cardiovascular changes independent of baseline risk. Where temporal information relative to MS diagnosis or treatment initiation is not reported, such estimates will be considered non-differentiated comorbidity burden rather than confirmed independent conditions.

Additional contextual factors will be considered when interpreting results. Reverse causality may occur when early manifestations of MS—such as mood disorders, fatigue, or migraine—are recorded as comorbidities rather than prodromal features of the disease. Ascertainment bias is likely, as children with MS undergo more frequent medical evaluations, increasing the detection of subclinical or mild conditions compared with the general pediatric population. Variability in data sources and diagnostic methods (e.g., ICD codes, registries, self-report, clinician-confirmed diagnoses) may introduce misclassification. Differences in health-care systems, screening practices, and treatment eras may further influence reported rates across regions and time periods.

Findings will therefore be framed as observed comorbidity patterns reflecting clinical practice and data collection contexts, rather than definitive causal relationships.

## 3. Discussion

This protocol outlines a systematic approach to quantifying the prevalence of comorbidities in POMS. While single-center and region-specific studies have provided estimates, a comprehensive synthesis at a global level remains lacking. By aggregating data from diverse settings, this systematic review will generate more reliable prevalence estimates to support clinical screening and management in pediatric MS.

Beyond prevalence, this review will also explore incidence, risk, and characteristics of comorbidities where data are available, analyzing potential variations by age, sex, study design, geographic region, and disease-modifying therapy use. Additionally, clinical and demographic characteristics of comorbidities will be examined, along with their potential impact on disease trajectory, treatment decisions, and quality of life. In cases where meta-analysis is not feasible, a narrative synthesis will highlight key insights into the relationship between comorbidities and pediatric MS outcomes.

By incorporating these broader dimensions, this review aims to provide a clearer understanding of the role of comorbidities in POMS. The findings will help clinicians anticipate comorbid conditions, refine treatment approaches, and improve patient monitoring. Ultimately, the evidence generated will contribute to better-informed clinical decisions, guide future research priorities, and enhance long-term outcomes for children and adolescents with MS.

## 4. Limitations

Several limitations should be considered when interpreting the findings of this review. First, the search strategy is centered on PubMed and Embase; although complemented by reference screening, citation tracking, conference checks, and targeted sensitivity searches, some relevant studies indexed exclusively in other databases may not be captured, which may limit the global representativeness of the evidence. Second, the review is restricted to publications in English, German, and French; therefore, relevant studies in other languages may have been missed, further constraining a truly global perspective. Third, the literature on pediatric MS comorbidities is expected to be heterogeneous in design, case definitions, and data sources and may limit the comparability of estimates and preclude meta-analysis for certain conditions. Fourth, many studies may rely on administrative codes or self-reported diagnoses, introducing potential misclassification bias. Fifth, some reported conditions may reflect treatment-related or prodromal manifestations rather than independent comorbidities; the lack of temporal information relative to MS diagnosis or steroid/DMT initiation may limit differentiation between these scenarios. Sixth, evidence on incidence and risk is likely to be more limited than prevalence, as these outcomes require specific study designs with appropriate denominators and well-defined comparator groups; variability in comparator selection may further restrict quantitative pooling. Seventh, grey literature sources such as conference abstracts often lack complete methodological details and denominators, limiting their suitability for quantitative synthesis. Finally, differences in health-care systems, diagnostic practices, and comorbidity ascertainment across regions may affect the generalizability of pooled estimates. These limitations will be considered when interpreting the results, and where pooling is not feasible, a narrative synthesis will be used to provide a balanced and transparent summary.

## Figures and Tables

**Table 1:** Comorbidity Categories of Interest in Pediatric-Onset Multiple Sclerosis (POMS).

**Comorbidity Category**	**Examples/Notes**
Vascular Disease and Risk Factors (Endocrine and Metabolic Disorders)	Hypertension, dyslipidemia, atherosclerosis, Type 2 diabetes, dysmetabolic syndrome X (Includes overlapping risk factors with autoimmune conditions)
Heart Disease	Congenital heart conditions, arrhythmias, heart failure
Mental, Behavioral, and Neurodevelopmental Disorders	Depression, anxiety, ADHD, autism spectrum disorders
Diseases of the Nervous System/Neurological Disorders	Epilepsy, migraine, peripheral neuropathies
Diseases of the Respiratory System	Asthma, chronic obstructive pulmonary disease (COPD)-like syndromes, cystic fibrosis (if relevant)
Diseases of the Digestive System	Inflammatory bowel disease (Crohn’s disease, ulcerative colitis), celiac disease
Neoplasms (Cancer)	Leukemia, lymphoma, solid tumors
Diseases of the Immune System/Autoimmune Disorders	Primary immunodeficiencies, other autoimmune conditions not otherwise classified
Diseases of the Musculoskeletal System and Connective Tissue	Juvenile rheumatoid arthritis, lupus, other connective tissue diseases
Diseases of the Blood and Blood-Forming Organs	Anemia, clotting disorders
Diseases of the Skin	Psoriasis, eczema, other chronic inflammatory skin disorders
Diseases of the Genitourinary System	Chronic kidney disease, nephrotic syndrome
Infections and Parasitic Diseases	Chronic viral infections (e.g., HIV, hepatitis), parasitic infections
Diseases of the Eye	Uveitis, retinitis

**Table 2:** Eligibility criteria in study selection.

**Criteria**	**Inclusion Criteria**	**Exclusion Criteria**
Population features – Disease definition *	Children and adolescents diagnosed with Multiple Sclerosis (pediatric MS). MS diagnosed in individuals younger than 18 years of age, according to the International Pediatric Multiple Sclerosis Study Group (IPMSSG) criteria, which include clinical and MRI evidence of central nervous system demyelination disseminated in time and space, not better explained by another disease process.	Studies focusing solely on adult MS populations, Other demyelinating diseases, Inability to extract data on pediatric MS in studies reporting a mixed sample of MS with other diseases or with adult MS
Comorbid conditions	Studies examining one or more of the specified 15 comorbidity categories. (The 15 comorbid conditions as specified by a health professional, through medical records/registries, self-administered questionnaires/reports, administered data, ICD codes, and/or databases).	Studies not focusing on the specified comorbidities
Intervention	Not applicable as this review focuses on observational data regarding comorbidities	Studies focusing on interventions not related to comorbidities
Study Design	Any type of observational study (including cross-sectional, case–control, and cohort designs) will be included. If observational data are insufficient, interventional studies (randomized controlled trials, non-randomized controlled trials, crossover trials) and long-term follow-up studies derived from prior interventional research will also be considered.	Narrative reviews, case reports/series, conference abstracts or articles with no primary data, editorials without data, studies that focus on therapeutic interventions, policy papers, study protocols without baseline data, animal studies, and pre-print studies
Sample size	No restriction	
Publication Type	Peer-reviewed articles, systematic reviews	Books, conference proceedings not peer-reviewed, unpublished manuscripts
Language	English, German, French	Non-English articles without available English translations

**Note:** To avoid violating independent assumptions, studies were included only once. When more than one article reports data from the same study population, the following hierarchy will be applied: (1) the report with the most complete comorbidity information, (2) the largest sample size, (3) the most recent dataset, and (4) the longest follow-up for incidence/risk outcomes. If different publications provide non-overlapping comorbidity data, relevant information will be extracted separately while avoiding double counting. * For a study to be included under the pediatric MS category, it must explicitly state that the population studied falls within the pediatric age range (up to 18 years old) or provide data that can be distinctly analyzed for this age group if mixed with adult populations.

**Table 3:** Sample of the extraction sheet.

**Field Number**	**Field Description**	**Data (Example/Notes)**
1	Study ID	[Unique Identifier]
2	Author(s)	
3	Year of Publication	
4	Country	
5	Study Design	
6	Sample Size	
7	Age Range	
8	Gender Distribution	
9	Inclusion Criteria	
10	Exclusion Criteria	
11	Diagnosis Criteria Used	
12	Disease Duration of MS	
13	Type of MS	
14	List of Comorbidities Studied	
15	Method of Comorbidity Diagnosis	
16	Prevalence Data	
17	Incidence Data	
18	Risk Data	
19	Statistical Methods Used	
20	Outcome Significance	*p*-values, confidence intervals
21	Adjustments Made in Analysis	
22	Summary of Major Findings	
23	Risk of Bias	
24	Conflicts of Interest	
25	Comments/Remarks	

## Data Availability

The original contributions presented in this study are included in the article/Supplementary Material. Further inquiries can be directed to the corresponding author.

## Supplementary Materials

The following supporting information can be downloaded at: https://www.mdpi.com/article/10.3390/children13020307/s1. Extraction Sheet S1; PRISMA Checklist S1; Table S1: Feasibility Check in MEDLINE (via PubMed) (10 February 2025); Table S2: Search strategy for MEDLINE (via PubMed); Table S3: Search strategy for Embase (produced by Elsevier).

## Abbreviations

AOMS: Adult-onset multiple sclerosis; ADEM: Acute disseminated encephalomyelitis; CI: Confidence interval; COPD: Chronic obstructive pulmonary disease; DMT: Disease-modifying therapy; ICD: International Classification of Diseases; IPMSSG: International Pediatric Multiple Sclerosis Study Group; JBI: Joanna Briggs Institute; MeSH: Medical Subject Headings; MS: Multiple sclerosis; OR: Odds ratio; OSF: Open Science Framework; POMS: Pediatric-onset multiple sclerosis; PRISMA: Preferred Reporting Items for Systematic Reviews and Meta-Analyses; PRISMA-P: Preferred Reporting Items for Systematic Reviews and Meta-Analyses for Protocols; QoL: Quality of life; RevMan: Review Manager; RR: Relative risk.
