# Supplementary material for: Prevalence, Incidence, and Risk of Different Comorbidity Categories in Pediatric Multiple Sclerosis: A Systematic Review and Meta-Analysis Protocol
Source: Children (Basel). 2026 Feb 23;13(2):307. doi: 10.3390/children13020307 (PMC12939965; doi:10.3390/children13020307)
Supplement: Supplementary file 1 [file children-13-00307-s001.zip › Supplementary file(tables).pdf]

Supplementary Table S1: Feasibility Check in MEDLINE (via PubMed) (10.02.25)	
Search combination	Hits
1+2	10,225
1+2+3	488
1+2+4	322
1+2+5	472
1+2+6	337
1+2+7	999
1+2+8	481
1+2+9	283
1+2+10	300
1+2+11	841
1+2+12	616
1+2+13	262
1+2+14	313
1+2+15	264
1+2+16	280
1+2+17	502
1+2+18	128
(1 AND 2) AND (3 OR 4 OR 5 OR...OR 18) (+ Extra filters)	3267 (2626 +extra filters)

Supplementary Table S2: Search strategy for MEDLINE (via PubMed)		
No.	Domain	Search terms in PubMed
1	Multiple Sclerosis (MS)	"Multiple Sclerosis"[MeSH] OR "Multiple Sclerosis"[Title/Abstract]
2	Pediatrics	"Pediatrics"[Mesh] OR "Child"[Mesh] OR "Adolescent"[Mesh] OR "Child, Preschool"[Mesh] OR "pediatric"[tiab] OR "child"[tiab] OR "adolescent"[tiab]

3	Comorbidity and related terms	"Comorbidity"[Mesh] OR "Multimorbidity"[Mesh] OR "co-morbid*"[tiab] OR "comorbid*"[tiab] OR "comorbid condition*"[tiab] OR "comorbid disease*"[tiab] OR "comorbid disorder*"[tiab] OR "comorbid health condition*"[tiab] OR "comorbid medical condition*"[tiab] OR "co-morbid condition*"[tiab] OR "co-morbid disease*"[tiab] OR "co-morbid disorder*"[tiab] OR "co-morbid health condition*"[tiab] OR "co-morbid medical condition*"[tiab] OR "multimorbid*"[tiab] OR "multi-morbid*"[tiab] OR "multiple condition*"[tiab] OR "multiple chronic condition*"[tiab] OR "multiple chronic disease*"[tiab] OR "multiple disease*"[tiab] OR "multiple diagnos*"[tiab] OR "multiple health problem*" OR "multiple illness*"[tiab] OR "multiple patholog*"[tiab] OR "multiple morbid*"[tiab] OR "multiple disorder*"[tiab] OR "multidisease*"[tiab] OR "multi-disease*"[tiab] OR "multipatholog*"[tiab] OR "multi-patholog*"[tiab] OR "pluripatholog*"[tiab] OR "concomitant disease*"[tiab] OR "concomitant illness*"[tiab] OR "concomitant disorder*"[tiab] OR "concomitant health problem*"[tiab] OR "concomitant medical condition*"[tiab] OR "co-occurring condition*"[tiab] OR "co-occurring disease*"[tiab] OR "coexisting condition* "[tiab] OR "co-existing condition*"[tiab] OR "coexisting disease*"[tiab] OR "coexisting disease*" [tiab] OR "coexisting health problem*"[tiab] OR "co-existing health problem*"[tiab] OR "coexistent disease*"[tiab] OR "coexistent chronic condition*"[tiab] OR "co-existent disease*"[tiab] OR "co-existent medical condition*"[tiab] OR "concurrent condition*"[tiab] OR "concurrent disease*"[tiab] OR "concurrent chronic condition*"[tiab] OR "concurrent chronic disease*"[tiab] OR "concurrent illness*"[tiab] OR "concurrent diagnos*"[tiab] OR "concurrent health problem*"[tiab] OR "concurrent chronic disorder*"[tiab] OR "concurrent disorder*"[tiab] OR "concurrent patholog*"[tiab] OR "concurrent morbid*"[tiab] OR "associated illness*"[tiab] OR "associated health problem*"[tiab] OR "associated morbid*"[tiab] OR "associated diagnos*"[tiab] OR "associated health condition*"[tiab] OR "associated medical condition*"[tiab]) OR "Overlapping conditions"[tiab]

4	Vascular Disease (Some diseases are not chronic, but they share characteristics with vascular risk factors and some overlap with autoimmune disorders.)	"Vascular Disease*"[tiab] "Aneurysm"[tiab] OR "Peripheral Arterial Disease*"[tiab] OR "Venous Insufficiency"[tiab] OR "Varicose Veins"[tiab] OR "Hypertension"[tiab] OR "High Blood Pressure"[tiab] OR "Stroke"[tiab] OR "Cerebrovascular Accident"[tiab] OR "Angina Pectoris"[tiab] OR "Chest Pain"[tiab] OR "Myocardial Infarction"[tiab] OR "Heart Attack"[tiab] OR "Deep Vein Thrombosis"[tiab] OR "Pulmonary Embolism"[tiab] OR "Raynaud's Phenomenon"[tiab] OR "Coronary Artery Disease*"[tiab] OR "Venous Thromboembolism"[tiab] OR "Carotid Artery Disease*"[tiab] OR "Lymphedema"[tiab] OR "Arteriovenous Malformation"[tiab] OR "Chronic Venous Insufficiency"[tiab] OR "Peripheral Vascular Dis*"[tiab] OR "Buerger's Disease*"[tiab] OR "Fibromuscular Dysplasia"[tiab] OR "Giant Cell Arteritis"[tiab] OR "Temporal Arteritis"[tiab] OR "Kawasaki Disease*"[tiab] OR "Phlebitis"[tiab] OR "Takayasu Arteritis"[tiab] OR "Vascular Dementia"[tiab] OR "Vascular Ehlers-Danlos Syndrome"[tiab] OR "Blood Vessel Disease*"[tiab] OR "Cerebral Amyloid Angiopathy"[tiab] OR "Coronary Vasospasm"[tiab] OR "Hemangioma"[tiab] OR "Hereditary Hemorrhagic Telangiectasia"[tiab] OR "Osler-Weber-Rendu syndrome"[tiab] OR "Hypotension"[tiab] OR "Low Blood Pressure"[tiab] OR "Intracranial Aneurysm"[tiab] OR "Lymphangioma"[tiab] OR "Antiphospholipid Syndrome"[tiab] OR "Renal Artery Disease*"[tiab] OR "Retinal Vasculopathy"[tiab] OR "Mesenteric Ischemia"[tiab]

5	Vascular Risk Factors (Endocrine and Metabolic Disorders)	"Metabolic Syndrome*"[tiab] OR "Insulin Resistance"[tiab] OR "Syndrome X"[tiab] OR "Dysmetabolic Syndrome X"[tiab] OR "Reaven Syndrome X"[tiab] OR "Metabolic Cardiovascular Syndrome"[tiab] OR "Cardiometabolic Syndrome*"[tiab]<br>OR<br>"Hypertension"[tiab] OR "High Blood Pressure*"[tiab]<br>OR<br>Hyperlipidemia*[tiab] OR Hyperlipemias[tiab] OR Dyslipidemia*[tiab] OR Hypertriglyceridemia*[tiab] OR Hypertriglyceridemic[tiab] OR Hypercholesterolemia*[tiab] OR "High Cholesterol Level*"[tiab] OR "Elevated Cholesterol"[tiab] OR "Elevated Cholesterol*"[tiab] OR Hypercholesteremia*[tiab] OR Hyperlipoproteinemia*[tiab] OR Dyslipoproteinemia*[tiab] OR "Lipid Metabolism Disease*"[tiab]<br>OR<br>"Diabetes Mellitus"[tiab] OR "Diabetes"[tiab] OR "Diabetes Insipidus"[tiab] OR "Prediabetic State"[tiab] OR "Scleredema Adultorum"[tiab] OR "Glucose Intolerance"[tiab] OR Gastroparesis[tiab] OR "Glucose Metabolic Disease*"[tiab] OR "Glucose Metabolism Disease*"[tiab] OR "Prediabetic Stat*"[tiab] OR Prediabetic[tiab] OR Prediabetes[tiab] OR "Latent Autoimmune Diabetes"[tiab] OR Leprechaunism*[tiab] OR "Rabson-Mendenhall Syndrome*"[tiab]<br>OR<br>"Smoking"[tiab] OR "Tobacco"[tiab] OR Cigar[tiab] OR Cigarette[tiab] OR pipe[tiab] OR Waterpipe[tiab] OR "Hookah Smoking"[tiab] OR "Herbal Smoking" [tiab] OR "E-Cigarette"[tiab] OR ECig[tiab] OR E-Cig[tiab] OR "E Cig"[tiab] OR Vaping*[tiab] OR Vape*[tiab]<br>OR<br>"Obesity"[tiab] OR Obesities[tiab] OR "Appetite Depressants"[tiab] OR "Anti-Obesity Agent*"[tiab] OR Bariatric*[tiab] OR Overweight[tiab] OR "Prader Willi Syndrome"[tiab] OR "Royer Syndrome"[tiab] OR<br>OR<br>Alcoholism[tiab] OR "Alcohol Dependence"[tiab] OR "Alcohol Addiction"[tiab] OR "Alcohol Abuse"[tiab]<br>OR<br>"Sedentary Lifestyle"[tiab] OR "Physical Inactivity"[tiab] OR "Life Style Induced Illness"[tiab]

6	Heart Disease	"Heart Diseases"[Mesh] OR "Cardiac Disease*"[tiab] OR "Heart Disease*"[tiab] OR "Heart Failure"[tiab] OR "Cardiomyopathy"[tiab] OR Arrhythmia*[tiab] OR "Heart Rhythm Disease*"[tiab] OR "Valvular Heart Disease*"[tiab] OR "Cardiac Arrest"[tiab] OR "Heart Valve Dis"[tiab] OR "Pericarditis"[tiab] OR "Endocarditis"[tiab] OR "Myocarditis"[tiab] OR "Sudden Cardiac Death"[tiab] OR "Bradyarrhythmia*"[tiab] OR "Tachyarrhythmia*"[tiab] OR "Congenital Heart disease*"[tiab] OR "Congenital Heart Defect*"[tiab] OR "Cardiac Hypertrophy"[tiab] OR "cardiac arrhythmia*"[tiab] OR "cardiac dysrhythmia"[tiab] OR "atrial fibrillation"[tiab] OR "sinus arrhythmia"[tiab] OR "atrial flutter"[tiab] OR bradycardia[tiab] OR "Brugada syndrome"[tiab] OR "heart block"[tiab] OR "long qt syndrome"[tiab] OR "parasystole"[tiab] OR "pre-excitation syndrome*"[tiab] OR "tachycardia*"[tiab] OR "ventricular fibrillation"[tiab] OR "ventricular flutter"[tiab] OR "cardiovascular disease"[tiab] OR "heart disease"[tiab] OR "Cardiomyopathy"[tiab] OR "coronary artery disease"[tiab] OR "acute coronary syndrome"[tiab] OR "coronary occlusion"[tiab] OR "coronary artery occlusion"[tiab] OR cardio[tiab] OR heart[tiab] OR cardiomyopath*[tiab] OR myocard[tiab] OR coronar*[tiab] OR vascula*[tiab] OR cardiac*[tiab]
7	Mental, Behavioral and Neurodevelopmental Disorders	"Mental Disorders"[Mesh] OR "alcohol-related disorders"[tiab] OR "drinking behavior"[tiab] OR alcoholic*[tiab] OR alcoholism[tiab] OR "alcohol abuse"[tiab] OR "alcohol use"[tiab] OR "alcohol misuse"[tiab] OR "alcohol problem"[tiab] OR "alcohol depend*"[tiab] OR "anxiety disorder"[tiab] OR "generalized anxiety disorder"[tiab] OR phobia[tiab] OR "panic disorder"[tiab] OR "obsessive compulsive disorder"[tiab] OR "posttraumatic stress disorder"[tiab] OR "bipolar disorder"[tiab] OR "manic depression"[tiab] OR "manic depressive disorder"[tiab] OR mania[tiab] OR "depressive disorder"[tiab] OR "major depression"[tiab] OR dysthymia[tiab] OR "depressive symptoms"[tiab] OR depression[tiab] OR depressed[tiab] OR psychosis[tiab] OR "psychotic disorders"[tiab] OR schizophrenia[tiab] OR "delusional disorder*"[tiab] OR psychotic*[tiab] OR "schizoaffective disorder"[tiab] OR "thought disturbances"[tiab] OR psychoses[tiab] OR "substance-related disorders"[tiab] OR addict*[tiab] OR abuse*[tiab] OR narcotic*[tiab] OR opiates[tiab] OR opioid[tiab] OR heroin[tiab] OR crack[tiab] OR cocaine[tiab] OR amphetamine*[tiab] OR marijuana[tiab] OR cannabis[tiab] OR phencyclidine[tiab] OR "street drugs"[tiab] OR "designer drugs"[tiab] OR "personality disorder*"[tiab] OR antisocial[tiab] OR "borderline disorder*"[tiab] OR histrionic[tiab] OR narcissistic[tiab] OR "obsessive compulsive"[tiab] OR paranoid[tiab] OR "passive aggressive"[tiab] OR sadomasochistic[tiab] OR schizoid[tiab] OR schizotypal[tiab]

8	Diseases of the Nervous System/ Neurological Disorders	"seizure*"[tiab] OR "epileps*"[tiab] OR "Pediatric Stroke"[tiab] OR "Cerebral Palsy"[tiab] OR "Neurofibromatosis"[tiab] OR "Tuberous Sclerosis"[tiab] OR "Rett Syndrome"[tiab] OR "Childhood Dystonia"[tiab] OR "Juvenile Parkinson's Disease"[tiab] OR "Myasthenia Gravis"[tiab] OR "Autism Spectrum Disorder"[tiab] OR "Attention Deficit Hyperactivity Disorder"[tiab] OR "Pediatric Migraine"[tiab] OR "Landau-Kleffner Syndrome"[tiab] OR "West Syndrome"[tiab] OR "Dravet Syndrome"[tiab] OR "Lennox-Gastaut Syndrome"[tiab] OR "Movement Disorder*"[tiab] OR "Myotonic Disorder*"[tiab] OR "Peripheral Nerve Disease*"[tiab]
9	Diseases of the Respiratory System	"Lung Diseases"[Mesh] OR "asthma"[tiab] OR "Chronic Obstructive Pulmonary Disease"[tiab] OR "pulmonary"[tiab] OR "pulmonary disease"[tiab] OR "lung"[tiab] OR "lung disease"[tiab] OR "bronchitis"[tiab] OR "emphysema"[tiab] OR ("Disease" AND "Lung")[tiab] OR ("Diseases" AND "Lung")[tiab] OR "Lung Disease"[tiab] OR "Pulmonary Disease"[tiab] OR ("Disease" AND "Pulmonary")[tiab] OR ("Diseases" AND "Pulmonary")[tiab] OR "Pulmonary Diseases"[tiab] OR "Respiratory Tract Diseases"[tiab] OR "Acute Chest Syndrome"[tiab] OR "alpha 1-Antitrypsin Deficiency"[tiab] OR "Cystic Fibrosis"[tiab] OR "Hemoptysis"[tiab] OR "Hepatopulmonary Syndrome"[tiab] OR ("Hypertension" AND "Pulmonary")[tiab] OR "Familial Primary Pulmonary Hypertension"[tiab] OR "Persistent Fetal Circulation Syndrome"[tiab] OR "Pulmonary Arterial Hypertension"[tiab] OR "Lung Abscess"[tiab] OR "Blastomycosis"[tiab] OR ("Pneumonia" AND "Pneumocystis")[tiab] OR "Pulmonary Aspergillosis"[tiab] OR "Sarcoidosis"[tiab] OR "Pulmonary"[tiab] OR ("Pulmonary Disease" AND "Chronic Obstructive")[tiab] OR "Pneumonia"[tiab] OR "Respiratory Distress Syndrome"[tiab] OR "Scimitar Syndrome"[tiab] OR "Solitary Pulmonary Nodule"[tiab]

10	Diseases of the Digestive System	"Gastrointestinal Diseases"[Mesh] OR "gastroesophageal reflux"[tiab] OR "GERD"[tiab] OR "GORD"[tiab] OR "heartburn"[tiab] OR "esophagitis"[tiab] OR "oesophagitis"[tiab] OR "liver disease"[tiab] OR "hepatitis"[tiab] OR "cirrhosis"[tiab] OR "fibrosis"[tiab] OR "fatty liver"[tiab] OR "alcoholic liver disease"[tiab] OR "alcoholic hepatitis"[tiab] OR "peptic ulcer"[tiab] OR "duodenal ulcer"[tiab] OR "gastroduodenal ulcer"[tiab] OR "gallbladder disease"[tiab] OR "cholelithiasis"[tiab] OR "choledocholithiasis"[tiab] OR "cholangitis"[tiab] OR "biliary"[tiab] OR "irritable bowel syndrome"[tiab] OR "irritable colon"[tiab] OR "dysphagia"[tiab] OR ("Disease" AND "Gastrointestinal")[tiab] OR ("Diseases" AND "Gastrointestinal")[tiab] OR "Gastrointestinal Disease"[tiab] OR "Gastrointestinal Disorders"[tiab] OR "Gastrointestinal Disorder"[tiab] OR "Functional Gastrointestinal Disorders"[tiab] OR "Functional Gastrointestinal Disorder"[tiab] OR ("Gastrointestinal Disorder" AND "Functional")[tiab] OR ("Gastrointestinal Disorders" AND "Functional")[tiab] OR "Cholera Infantum"[tiab] OR "Esophageal Diseases"[tiab] OR "Barrett Esophagus"[tiab] OR "Deglutition Disorders"[tiab] OR (Diverticulosis AND "Esophageal")[tiab] OR "Gastric Varices"[tiab] OR "Esophageal Atresia"[tiab] OR "Esophageal Cyst"[tiab] OR "Esophageal Fistula"[tiab] OR "Esophageal Neoplasms"[tiab] OR "Esophageal Perforation"[tiab] OR "Esophageal Stenosis"[tiab] OR "Esophagitis"[tiab] OR "Appendicitis"[tiab] OR "Cholera Morbus"[tiab] OR "Diverticular Diseases"[tiab] OR "Dysentery"[tiab] OR "Enteritis"[tiab] OR "Enterocolitis"[tiab] OR "Gastritis"[tiab] OR "Mucositis"[tiab] OR "Proctitis"[tiab] OR "Inflammatory Bowel Diseases"[tiab] OR "Hematemesis"[tiab] OR "Melena"[tiab] OR "Peptic Ulcer Hemorrhage"[tiab] OR "Esophageal Neoplasms"[tiab] OR "Gastrointestinal Stromal Tumors"[tiab] OR "Intestinal Neoplasms"[tiab] OR "Stomach Neoplasms"[tiab] OR "Zollinger-Ellison Syndrome"[tiab] OR "Cecal Diseases"[tiab] OR "Colonic Diseases"[tiab] OR "Duodenal Diseases"[tiab] OR "Dysentery"[tiab] OR "Enteritis"[tiab] OR "Enterocolitis"[tiab] OR "HIV Enteropathy"[tiab] OR "Ileal Diseases"[tiab] OR "Intestinal Atresia"[tiab] OR ("Intestinal Diseases" AND "Parasitic")[tiab] OR "Intestinal Fistula"[tiab] OR "Intestinal Neoplasms"[tiab] OR "Intestinal Obstruction"[tiab] OR "Intestinal Perforation"[tiab] OR "Intestinal Polyposis"[tiab] OR "Jejunal Diseases"[tiab] OR "Malabsorption Syndromes"[tiab] OR "Mesenteric Ischemia"[tiab] OR "Mesenteric Vascular Occlusion"[tiab] OR "Pneumatosis Cystoides Intestinalis"[tiab] OR "Protein-Losing Enteropathies"[tiab] OR "Rectal Diseases"[tiab] OR "Rumination Syndrome"[tiab] OR "Achlorhydria"[tiab] OR (Diverticulosis AND "Stomach")[tiab] OR "Duodenogastric Reflux"[tiab] OR "Gastric Antral Vascular Ectasia"[tiab] OR "Gastric Dilatation"[tiab] OR "Gastric Outlet

		Obstruction"[tiab] OR "Gastritis"[tiab] OR "Gastroparesis"[tiab] OR "Peptic Ulcer"[tiab] OR "Postgastrectomy Syndromes"[tiab] OR "Stomach Neoplasms"[tiab] OR "Stomach Rupture"[tiab] OR "Stomach Volvulus"[tiab] OR "Zollinger-Ellison Syndrome"[tiab] OR "Visceral Prolapse"[tiab] OR "Reflux"[tiab] OR "Appendicitis"[tiab] OR "Barrett's Esophagus"[tiab] OR "Celiac Disease"[tiab] OR "Celiac"[tiab] OR "Constipation"[tiab] OR "Crohn's Disease"[tiab] OR "Dumping Syndrome"[tiab] OR "Gallstones"[tiab] OR "Indigestion"[tiab] OR "Dyspepsia"[tiab] OR "Hernia"[tiab] OR "Intestinal Pseudo-obstruction"[tiab] OR "obstruction"[tiab] OR "Menetrier's Disease"[tiab] OR "Peptic Ulcers"[tiab] OR "ulcer"[tiab] OR "Short Bowel Syndrome"[tiab] OR "Ulcerative Colitis"[tiab]
11	Neoplasms (Cancer)	"Neoplasms"[Mesh] OR "Cancer"[tiab] OR "carcinoma"[tiab] OR "neoplasia"[tiab] OR "tumor"[tiab] OR "neoplasm"[tiab] OR "maligna*"[tiab] AND "German"[Language] AND "English"[Language] AND "French"[Language]

12	Diseases of the Immune System/ Autoimmune Disorders	"hypothyroidism"[tiab] OR "myxedema"[tiab] OR "hyperthyroidism"[tiab] OR "thyrotoxicosis"[tiab] OR "Graves disease"[tiab] OR "thyroiditis"[tiab] OR "Hashimoto's"[tiab] OR "rheumatoid arthritis"[tiab] OR "Felty syndrome"[tiab] OR "Rheumatoid nodule"[tiab] OR "Rheumatoid vasculitis"[tiab] OR "adult onset Still's disease"[tiab] OR "dermatomyositis"[tiab] OR "polymyositis"[tiab] OR "idiopathic inflammatory myopathies"[tiab] OR "myositis"[tiab] OR "ankylosing spondylitis"[tiab] OR "spondylitis ankylosing"[tiab] OR "Rheumatoid spondylitis"[tiab] OR "vertebral ankylosis"[tiab] OR "spondyloarthropathy"[tiab] OR "bechterew*"[tiab] OR "Bechterew's disease"[tiab] OR "Marie-Strumpell*"[tiab] OR "Inflammatory bowel disease"[tiab] OR "Crohn's"[tiab] OR "ulcerative colitis"[tiab] OR "uveitis"[tiab] OR "uveiti*"[tiab] OR "panuveitis"[tiab] OR "iridocyclitis"[tiab] OR "iridocycliti*"[tiab] OR "anterior scleritis"[tiab] OR "iritis"[tiab] OR "iriti*"[tiab] OR "pars planitis"[tiab] OR "Lupus"[tiab] OR "Lupus erythematosus"[tiab] OR "Systemic lupus erythematosus"[tiab] OR "Lupus vulgaris"[tiab] OR "psoriasis"[tiab] OR "palmoplantaris pustulosis"[tiab] OR "pustular psoriasis"[tiab] OR "pemphigus"[tiab] OR "pemphigus vulgaris"[tiab] OR "pemphigus foliaceus"[tiab] OR "primary biliary cirrhosis"[tiab] OR "liver cirrhosis"[tiab] OR "bullous pemphigoid"[tiab] OR "pemphigoid"[tiab] OR "myasthenia"[tiab] OR "myasthenia gravis"[tiab] OR "anti-acetylcholine receptor antibody"[tiab] OR "anti-muscle specific kinase antibody"[tiab] OR "seronegative MG"[tiab] OR "myasthenia gravis"[tiab] OR "myasthenia"[tiab] OR "congenital myasthenic syndrome"[tiab] OR "systemic sclerosis"[tiab] OR "scleroderma"[tiab] OR "pernicious anaemia"[tiab] OR "atrophic gastritis"[tiab] OR "type A gastritis"[tiab] OR "macrocytic anaemia"[tiab] OR "cobalamin deficiency"[tiab] OR "vitamin B12 deficiency"[tiab] OR "gastritis"[tiab] OR "idiopathic thrombocytopenic purpura"[tiab] OR "thrombocytopeni*"[tiab] OR "Addison's disease"[tiab] OR "Addison disease"[tiab] OR "primary adrenal insufficiency"[tiab] OR "primary adrenocortical insufficiency"[tiab] OR "adrenal-Cortex-Diseases"[tiab] OR "adrenal-Gland-Diseases"[tiab] OR "adrenal-Insufficiency"[tiab] OR "Wegener Granulomatosis"[tiab] OR "vasculitis"[tiab] OR "wegener vascul*"[tiab] OR "small vessel vascul*"[tiab] OR "celiac"[tiab] OR "sprue"[tiab] OR "gluten sensitive enteropathy"[tiab] OR "villous atrophy"[tiab] OR "antigliadin"[tiab] OR "endomyseal"[tiab] OR "tissue transglutaminase"[tiab] OR "Alopecia areata"[tiab] OR "Alopecia totalis"[tiab] OR "Alopecia universalis"[tiab] OR "Ophiasis"[tiab] OR "non-scarring hair loss"[tiab] OR "vitiligo"[tiab] OR "leukoderma"[tiab] OR "leucoderma"[tiab] OR "Sjogren's syndrome"[tiab]

13	Diseases of the Musculoskeletal System and Connective Tissue	"gout"[tiab] OR "gouty arthritis"[tiab] OR "arthritis"[tiab] OR "osteoarthritis"[tiab] OR "hip osteoarthritis"[tiab] OR "knee osteoarthritis"[tiab] OR "spine osteoarthritis"[tiab] OR "fibromyalgia"[tiab] OR "fibromyositis"[tiab] OR "fibrositis"[tiab] OR "Bone Disease*"[tiab] OR "Coxa Magna"[tiab] OR "Coxa Valga"[tiab] OR "Osteitis"[tiab] OR "Osteochondritis"[tiab] OR "Osteochondrosis"[tiab] OR "Osteonecrosis"[tiab] OR "Spinal Disease*"[tiab] OR "Cartilage Disease*"[tiab] OR "Fasciitis"[tiab] OR "Foot Deformities"[tiab] OR "Foot Deformity"[tiab] OR "Hand Deformities"[tiab] OR "Hand Deformity"[tiab] OR "Jaw Disease*"[tiab] OR "Mandibular Disease*"[tiab] OR "Maxillary Disease*"[tiab] OR "Ankylosis"[tiab] OR "Arthralgia"[tiab] OR "Arthritis"[tiab] OR "Arthrogryposis"[tiab] OR "Bursitis"[tiab] OR "Contracture"[tiab] OR "Femoracetabular Impingement"[tiab] OR "Hallux Limitus"[tiab] OR "Hallux Rigidus"[tiab] OR "Hemarthrosis"[tiab] OR "Hydrarthrosis"[tiab] OR "Osteoarthropathy"[tiab] OR "Synovitis"[tiab] OR "Arthrogryposis"[tiab] OR "Myositis"[tiab] OR "Myotonic Disorders"[tiab] OR "Myotonic Disorder"[tiab] OR "Myotoxicity"[tiab] OR "Arthrogryposis"[tiab] OR "Campomelic Dysplasia"[tiab] OR "Synostosis"[tiab] OR "Pectus Carinatum"[tiab] OR "Gastroschisis"[tiab] OR "Craniofacial Abnormalities"[tiab] OR "Arthritis"[tiab] OR "Juvenile Arthritis"[tiab] OR "Rheumatoid Arthritis"[tiab] OR "Sternocostoclavicular Hyperostosis"[tiab] OR "Osteoarthritis"[tiab] OR "Polymyalgia Rheumatica"[tiab] OR "Rheumatic Fever"[tiab]
14	Diseases of the Blood and Blood-forming Organs	"Hematologic Diseases"[Mesh] OR "Anemia"[tiab] OR "Hemoglobinopathies"[tiab] OR "Thalassemia"[tiab] OR "Sickle Cell Disease"[tiab] OR "Hemophilia"[tiab] OR "Leukemia"[tiab] OR "Lymphoma"[tiab] OR "Myelodysplastic Syndromes"[tiab] OR "Myeloproliferative Disorders"[tiab] OR "Platelet Disorders"[tiab] OR "Hemostatic Disorders"[tiab] OR "Coagulation Disorders"[tiab] OR "Bleeding Disorders"[tiab] OR "Blood Coagulation Disorders"[tiab] OR "Iron Overload"[tiab] OR "Hemochromatosis"[tiab] OR "Polycythemia Vera"[tiab] OR "Leukopenia"[tiab] OR "Thrombocytopenia"[tiab] OR "Thrombosis"[tiab] OR "Hemostasis Disorders"[tiab] OR "Blood Transfusion"[tiab] OR "Bone Marrow Diseases"[tiab] OR "Aplastic Anemia"[tiab] OR "Lymphadenopathy"[tiab] OR "Multiple Myeloma"[tiab] OR "Blood Platelet Disorders"[tiab] OR "Blood Protein Disorders"[tiab] OR "Hematopoietic Stem Cell Transplantation"[tiab]

15	Diseases of the Skin	"Skin Diseases"[Mesh] OR "Dermatitis"[tiab] OR "Eczema"[tiab] OR "Psoriasis"[tiab] OR "Acne"[tiab] OR "Skin Neoplasms"[tiab] OR "Melanoma"[tiab] OR "Basal Cell Carcinoma"[tiab] OR "Squamous Cell Carcinoma"[tiab] OR "Urticaria"[tiab] OR "Hives"[tiab] OR "Alopecia"[tiab] OR "Hair Loss"[tiab] OR "Vitiligo"[tiab] OR "Rosacea"[tiab] OR "Impetigo"[tiab] OR "Fungal Skin Infections"[tiab] OR "Tinea"[tiab] OR "Scabies"[tiab] OR "Lichen Planus"[tiab] OR "Bullous Pemphigoid"[tiab] OR "Pemphigus"[tiab] OR "Herpes Zoster"[tiab] OR "Shingles"[tiab] OR "Leprosy"[tiab] OR "Hansen's Disease"[tiab] OR "Skin Ulcer"[tiab] OR "Decubitus Ulcer"[tiab] OR "Cellulitis"[tiab] OR "Erythema"[tiab] OR "Rash"[tiab] OR "Dermatologic Agents"[tiab] OR "Skin Care"[tiab] OR "Skin Aging"[tiab] OR "Wound Healing"[tiab] OR "Burns"[tiab] OR "Frostbite"[tiab] OR "Pressure Ulcer"[tiab] OR "Sunburn"[tiab] OR "Pigmentation Disorders"[tiab] OR "Melasma"[tiab] OR "Hyperpigmentation"[tiab] OR "Hypopigmentation"[tiab]
16	Diseases of the Genitourinary System	"Urologic Diseases"[Mesh] OR "Genital Diseases, Male"[Mesh] OR "Genital Diseases, Female"[Mesh] OR "Kidney Diseases"[tiab] OR "Urinary Tract Infections"[tiab] OR "UTI"[tiab] OR "Bladder Diseases"[tiab] OR "Cystitis"[tiab] OR "Urethritis"[tiab] OR "Prostatitis"[tiab] OR "Erectile Dysfunction"[tiab] OR "Prostate Cancer"[tiab] OR "Bladder Cancer"[tiab] OR "Kidney Stones"[tiab] OR "Nephrolithiasis"[tiab] OR "Urinary Incontinence"[tiab] OR "Overactive Bladder"[tiab] OR "Interstitial Cystitis"[tiab] OR "Pelvic Organ Prolapse"[tiab] OR "Endometriosis"[tiab] OR "Polycystic Ovary Syndrome"[tiab] OR "PCOS"[tiab] OR "Uterine Fibroids"[tiab] OR "Menstrual Disorders"[tiab] OR "Ovarian Cysts"[tiab] OR "Gynecologic Cancer"[tiab] OR "Cervical Cancer"[tiab] OR "Ovarian Cancer"[tiab] OR "Endometrial Cancer"[tiab] OR "Pelvic Inflammatory Disease"[tiab] OR "PID"[tiab] OR "Sexually Transmitted Diseases"[tiab] OR "HIV/AIDS"[tiab] OR "Genital Herpes"[tiab] OR "Gonorrhea"[tiab] OR "Chlamydia"[tiab] OR "Syphilis"[tiab] OR "Human Papillomavirus"[tiab] OR OR "Urological Agents"[tiab] OR "Renal Failure"[tiab] OR "Chronic Kidney Disease"[tiab] OR "Acute Kidney Injury"[tiab]

17	Infections and Parasitic Diseases	"Communicable Diseases"[Mesh] OR "Parasitic Diseases"[Mesh] OR "Bacterial Infections"[tiab] OR "Viral Infections"[tiab] OR "Fungal Infections"[tiab] OR "Protozoan Infections"[tiab] OR "Helminthiasis"[tiab] OR "Worm Infections"[tiab] OR "Malaria"[tiab] OR "Tuberculosis"[tiab] OR "HIV"[tiab] OR "AIDS"[tiab] OR "Hepatitis"[tiab] OR "Influenza"[tiab] OR "COVID-19"[tiab] OR "SARS-CoV-2"[tiab] OR "Zika Virus"[tiab] OR "Dengue"[tiab] OR "Chikungunya"[tiab] OR "Lyme Disease"[tiab] OR "Syphilis"[tiab] OR "Gonorrhea"[tiab] OR "Chlamydia"[tiab] OR "Herpes Simplex"[tiab] OR "Herpes Zoster"[tiab] OR "Candidiasis"[tiab] OR "Aspergillosis"[tiab] OR "Cryptococcosis"[tiab] OR "Schistosomiasis"[tiab] OR "Leishmaniasis"[tiab] OR "Toxoplasmosis"[tiab] OR "Trichomoniasis"[tiab] OR "Giardiasis"[tiab] OR "Amoebiasis"[tiab] OR "Hookworm Infection"[tiab] OR "Ascariasis"[tiab] OR "Filariasis"[tiab] OR "Onchocerciasis"[tiab] OR "Trachoma"[tiab] OR "Antimicrobial Resistance"[tiab] OR "Antibiotic Resistance"[tiab]
18	Diseases Of the Eye	"Cataract"[tiab] OR "Glaucoma"[tiab] OR "Macular Degeneration"[tiab] OR "Diabetic Retinopathy"[tiab] OR "Uveitis"[tiab] OR "Conjunctivitis"[tiab] OR "Pink Eye"[tiab] OR "Corneal Diseases"[tiab] OR "Keratitis"[tiab] OR "Dry Eye Syndromes"[tiab] OR "Strabismus"[tiab] OR "Crossed Eyes"[tiab] OR "Amblyopia"[tiab] OR "Lazy Eye"[tiab] OR "Blepharitis"[tiab] OR "Color Vision Defects"[tiab] OR "Night Blindness"[tiab] OR "Refractive Errors"[tiab] OR "Myopia"[tiab] OR "Hyperopia"[tiab] OR "Astigmatism"[tiab] OR "Presbyopia"[tiab] OR "Eye Infections"[tiab] OR "Ocular Hypertension"[tiab] OR "Retinopathy of Prematurity"[tiab] OR "Vitreous Detachment"[tiab] OR "Retinal Detachment"[tiab]
	Extra filter	NOT "Case Reports"[Publication Type] NOT "Editorial"[Publication Type] NOT "Letter"[Publication Type] NOT "News"[Publication Type] AND "Humans"[Mesh] AND ("English"[Language] AND "German"[Language] AND "French"[Language])

(((((("Comorbidity"[MeSH Terms] OR "Multimorbidity"[MeSH Terms] OR "co morbid*"[Title/Abstract] OR "comorbid*"[Title/Abstract] OR "comorbid condition*"[Title/Abstract] OR "comorbid disease*"[Title/Abstract] OR "comorbid disorder*"[Title/Abstract] OR "comorbid health condition*"[Title/Abstract] OR "comorbid medical condition*"[Title/Abstract] OR "co morbid condition*"[Title/Abstract] OR "co morbid disease*"[Title/Abstract] OR "co morbid disorder*"[Title/Abstract] OR "co morbid health condition*"[Title/Abstract] OR "co morbid medical condition*"[Title/Abstract] OR "multimorbid*"[Title/Abstract] OR "multi morbid*"[Title/Abstract] OR "multiple condition*"[Title/Abstract] OR "multiple chronic condition*"[Title/Abstract] OR "multiple chronic disease*"[Title/Abstract] OR "multiple disease*"[Title/Abstract] OR "multiple diagnos*"[Title/Abstract] OR "multiple health problem*"[All Fields] OR "multiple illness*"[Title/Abstract] OR "multiple patholog*"[Title/Abstract] OR "multiple morbid*"[Title/Abstract] OR "multiple disorder*"[Title/Abstract] OR "multidisease*"[Title/Abstract] OR "multi disease*"[Title/Abstract] OR "multipatholog*"[Title/Abstract] OR "multi patholog*"[Title/Abstract] OR "pluripatholog*"[Title/Abstract] OR "concomitant disease*"[Title/Abstract] OR "concomitant illness*"[Title/Abstract] OR "concomitant disorder*"[Title/Abstract] OR "concomitant health problem*"[Title/Abstract] OR "concomitant medical condition*"[Title/Abstract] OR "co occurring condition*"[Title/Abstract] OR "co occurring disease*"[Title/Abstract] OR "coexisting condition*"[Title/Abstract] OR "co existing condition*"[Title/Abstract] OR "coexisting disease*"[Title/Abstract] OR "coexisting disease*"[Title/Abstract] OR "coexisting health problem*"[Title/Abstract] OR "co existing health problem*"[Title/Abstract] OR "coexistent disease*"[Title/Abstract] OR "coexistent chronic condition*"[Title/Abstract] OR "co existent disease*"[Title/Abstract] OR "co existent medical condition*"[Title/Abstract] OR "concurrent condition*"[Title/Abstract] OR "concurrent disease*"[Title/Abstract] OR "concurrent chronic condition*"[Title/Abstract] OR "concurrent chronic disease*"[Title/Abstract] OR "concurrent illness*"[Title/Abstract] OR "concurrent diagnos*"[Title/Abstract] OR "concurrent health problem*"[Title/Abstract] OR "concurrent chronic disorder*"[Title/Abstract] OR "concurrent disorder*"[Title/Abstract] OR "concurrent patholog*"[Title/Abstract] OR "concurrent morbid*"[Title/Abstract] OR "associated illness*"[Title/Abstract] OR "associated health problem*"[Title/Abstract] OR "associated morbid*"[Title/Abstract] OR "associated diagnos*"[Title/Abstract] OR "associated health condition*"[Title/Abstract] OR "associated medical condition*"[Title/Abstract] OR "Overlapping conditions"[Title/Abstract] OR (("vascular disease*"[Title/Abstract] AND "Aneurysm"[Title/Abstract]) OR "peripheral arterial disease*"[Title/Abstract] OR "Venous Insufficiency"[Title/Abstract] OR "Varicose Veins"[Title/Abstract] OR "Hypertension"[Title/Abstract] OR "High Blood Pressure"[Title/Abstract] OR "Stroke"[Title/Abstract] OR "Cerebrovascular Accident"[Title/Abstract] OR "Angina Pectoris"[Title/Abstract] OR "Chest Pain"[Title/Abstract] OR "Myocardial Infarction"[Title/Abstract] OR "Heart Attack"[Title/Abstract] OR "Deep Vein Thrombosis"[Title/Abstract] OR "Pulmonary Embolism"[Title/Abstract] OR "Raynaud's Phenomenon"[Title/Abstract] OR "coronary artery disease*"[Title/Abstract] OR "Venous Thromboembolism"[Title/Abstract] OR "carotid artery disease*"[Title/Abstract] OR "Lymphedema"[Title/Abstract] OR "Arteriovenous Malformation"[Title/Abstract] OR "Chronic Venous Insufficiency"[Title/Abstract] OR "peripheral vascular dis*"[Title/Abstract] OR "buerger s disease*"[Title/Abstract] OR "Fibromuscular Dysplasia"[Title/Abstract] OR "Giant Cell Arteritis"[Title/Abstract] OR "Temporal Arteritis"[Title/Abstract] OR "kawasaki disease*"[Title/Abstract] OR "Phlebitis"[Title/Abstract] OR "Takayasu Arteritis"[Title/Abstract] OR "Vascular Dementia"[Title/Abstract] OR "Vascular Ehlers-Danlos Syndrome"[Title/Abstract] OR "blood vessel disease*"[Title/Abstract] OR "Cerebral Amyloid Angiopathy"[Title/Abstract] OR "Coronary Vasospasm"[Title/Abstract] OR "Hemangioma"[Title/Abstract] OR "Hereditary Hemorrhagic Telangiectasia"[Title/Abstract] OR "Osler-Weber-Rendu syndrome"[Title/Abstract] OR "Hypotension"[Title/Abstract] OR "Low Blood Pressure"[Title/Abstract] OR "Intracranial Aneurysm"[Title/Abstract] OR "Lymphangioma"[Title/Abstract] OR "Antiphospholipid Syndrome"[Title/Abstract] OR "renal artery disease*"[Title/Abstract] OR "Retinal Vasculopathy"[Title/Abstract] OR "Mesenteric Ischemia"[Title/Abstract]) OR ("metabolic syndrome*"[Title/Abstract] OR "Insulin Resistance"[Title/Abstract] OR "Syndrome X"[Title/Abstract] OR "Dysmetabolic Syndrome X"[Title/Abstract] OR "Reaven Syndrome X"[Title/Abstract] OR "Metabolic Cardiovascular Syndrome"[Title/Abstract] OR "cardiometabolic syndrome*"[Title/Abstract] OR "Hypertension"[Title/Abstract] OR "high blood pressure*"[Title/Abstract] OR "hyperlipidemia*"[Title/Abstract] OR "Hyperlipemias"[Title/Abstract] OR "dyslipidemia*"[Title/Abstract] OR "hypertriglyceridemia*"[Title/Abstract] OR "Hypertriglyceridemic"[Title/Abstract] OR "hypercholesterolemia*"[Title/Abstract] OR "high cholesterol level*"[Title/Abstract] OR "Elevated Cholesterol"[Title/Abstract] OR "elevated cholesterol*"[Title/Abstract] OR "hypercholesteremia*"[Title/Abstract] OR "hyperlipoproteinemia*"[Title/Abstract] OR "dyslipoproteinemia*"[Title/Abstract] OR "lipid metabolism disease*"[Title/Abstract] OR "Diabetes Mellitus"[Title/Abstract] OR "Diabetes"[Title/Abstract] OR "Diabetes Insipidus"[Title/Abstract] OR "Prediabetic State"[Title/Abstract] OR "Scleredema Adultorum"[Title/Abstract] OR "Glucose Intolerance"[Title/Abstract] OR "Gastroparesis"[Title/Abstract] OR "glucose metabolic disease*"[Title/Abstract] OR "glucose metabolism disease*"[Title/Abstract] OR "prediabetic stat*"[Title/Abstract] OR "Prediabetic"[Title/Abstract] OR "Prediabetes"[Title/Abstract] OR "Latent Autoimmune Diabetes"[Title/Abstract] OR "leprechaunism*"[Title/Abstract] OR "rabson mendenhall syndrome*"[Title/Abstract] OR "Smoking"[Title/Abstract] OR "Tobacco"[Title/Abstract] OR "Cigar"[Title/Abstract] OR "Cigarette"[Title/Abstract] OR "pipe"[Title/Abstract] OR "Waterpipe"[Title/Abstract] OR "Hookah Smoking"[Title/Abstract] OR "Herbal Smoking"[Title/Abstract] OR "E-Cigarette"[Title/Abstract] OR "ECig"[Title/Abstract] OR "e cig"[Title/Abstract] OR "e cig"[Title/Abstract] OR "vaping*"[Title/Abstract] OR "vape*"[Title/Abstract] OR "Obesity"[Title/Abstract] OR "Obesities"[Title/Abstract] OR "Appetite Depressants"[Title/Abstract] OR "anti obesity agent*"[Title/Abstract] OR "bariatric*"[Title/Abstract] OR "Overweight"[Title/Abstract] OR "Prader Willi Syndrome"[Title/Abstract] OR "Royer Syndrome"[Title/Abstract] OR "Alcoholism"[Title/Abstract] OR "Alcohol Dependence"[Title/Abstract] OR "Alcohol Addiction"[Title/Abstract] OR "Alcohol Abuse"[Title/Abstract] OR "Sedentary Lifestyle"[Title/Abstract] OR "Physical Inactivity"[Title/Abstract] OR "Life Style Induced Illness"[Title/Abstract]) OR ("Heart Diseases"[MeSH Terms] OR "cardiac disease*"[Title/Abstract] OR "heart disease*"[Title/Abstract] OR "Heart Failure"[Title/Abstract] OR "Cardiomyopathy"[Title/Abstract] OR "arrhythmia*"[Title/Abstract] OR "heart rhythm disease*"[Title/Abstract] OR "valvular heart disease*"[Title/Abstract] OR "Cardiac Arrest"[Title/Abstract] OR "Heart Valve Dis"[Title/Abstract] OR "Pericarditis"[Title/Abstract] OR "Endocarditis"[Title/Abstract] OR "Myocarditis"[Title/Abstract] OR "Sudden Cardiac Death"[Title/Abstract] OR "bradyarrhythmia*"[Title/Abstract] OR "tachyarrhythmia*"[Title/Abstract] OR "congenital heart disease*"[Title/Abstract] OR "congenital heart defect*"[Title/Abstract] OR "Cardiac Hypertrophy"[Title/Abstract] OR "cardiac arrhythmia*"[Title/Abstract] OR "cardiac dysrhythmia"[Title/Abstract] OR "atrial fibrillation"[Title/Abstract] OR "sinus arrhythmia"[Title/Abstract] OR "atrial flutter"[Title/Abstract] OR "bradycardia"[Title/Abstract] OR "Brugada syndrome"[Title/Abstract] OR "heart block"[Title/Abstract] OR "long qt syndrome"[Title/Abstract] OR "parasystole"[Title/Abstract] OR "pre excitation syndrome*"[Title/Abstract] OR "tachycardia*"[Title/Abstract] OR "ventricular fibrillation"[Title/Abstract] OR "ventricular flutter"[Title/Abstract] OR "cardiovascular disease"[Title/Abstract] OR "heart disease"[Title/Abstract] OR "Cardiomyopathy"[Title/Abstract] OR "coronary artery disease"[Title/Abstract] OR "acute coronary syndrome"[Title/Abstract] OR "coronary occlusion"[Title/Abstract] OR "coronary artery occlusion"[Title/Abstract] OR "cardio"[Title/Abstract] OR "heart"[Title/Abstract] OR "cardiomyopath*"[Title/Abstract] OR "myocard"[Title/Abstract] OR "coronar*"[Title/Abstract] OR "vascula*"[Title/Abstract] OR "cardiac*"[Title/Abstract]) OR ("Mental Disorders"[MeSH Terms] OR "alcohol-related disorders"[Title/Abstract] OR "drinking behavior"[Title/Abstract] OR

"alcoholic*"[Title/Abstract] OR "Alcoholism"[Title/Abstract] OR "Alcohol Abuse"[Title/Abstract] OR "alcohol use"[Title/Abstract] OR "alcohol misuse"[Title/Abstract] OR "alcohol problem"[Title/Abstract] OR "alcohol depend*"[Title/Abstract] OR "anxiety disorder"[Title/Abstract] OR "generalized anxiety disorder"[Title/Abstract] OR "phobia"[Title/Abstract] OR "panic disorder"[Title/Abstract] OR "obsessive compulsive disorder"[Title/Abstract] OR "posttraumatic stress disorder"[Title/Abstract] OR "bipolar disorder"[Title/Abstract] OR "manic depression"[Title/Abstract] OR "manic depressive disorder"[Title/Abstract] OR "mania"[Title/Abstract] OR "depressive disorder"[Title/Abstract] OR "major depression"[Title/Abstract] OR "dysthymia"[Title/Abstract] OR "depressive symptoms"[Title/Abstract] OR "depression"[Title/Abstract] OR "depressed"[Title/Abstract] OR "psychosis"[Title/Abstract] OR "psychotic disorders"[Title/Abstract] OR "schizophrenia"[Title/Abstract] OR "delusional disorder*"[Title/Abstract] OR "psychotic*"[Title/Abstract] OR "schizoaffective disorder"[Title/Abstract] OR "thought disturbances"[Title/Abstract] OR "psychoses"[Title/Abstract] OR "substance-related disorders"[Title/Abstract] OR "addict*"[Title/Abstract] OR "abuse*"[Title/Abstract] OR "narcotic*"[Title/Abstract] OR "opiates"[Title/Abstract] OR "opioid"[Title/Abstract] OR "heroin"[Title/Abstract] OR "crack"[Title/Abstract] OR "cocaine"[Title/Abstract] OR "amphetamine*"[Title/Abstract] OR "marijuana"[Title/Abstract] OR "cannabis"[Title/Abstract] OR "phencyclidine"[Title/Abstract] OR "street drugs"[Title/Abstract] OR "designer drugs"[Title/Abstract] OR "personality disorder*"[Title/Abstract] OR "antisocial"[Title/Abstract] OR "borderline disorder*"[Title/Abstract] OR "histrionic"[Title/Abstract] OR "narcissistic"[Title/Abstract] OR "obsessive compulsive"[Title/Abstract] OR "paranoid"[Title/Abstract] OR "passive aggressive"[Title/Abstract] OR "sadomasochistic"[Title/Abstract] OR "schizoid"[Title/Abstract] OR "schizotypal"[Title/Abstract]) OR ("seizure*"[Title/Abstract] OR "epileps*"[Title/Abstract] OR "Pediatric Stroke"[Title/Abstract] OR "Cerebral Palsy"[Title/Abstract] OR "Neurofibromatosis"[Title/Abstract] OR "Tuberous Sclerosis"[Title/Abstract] OR "Rett Syndrome"[Title/Abstract] OR "Childhood Dystonia"[Title/Abstract] OR "Juvenile Parkinson's Disease"[Title/Abstract] OR "Myasthenia Gravis"[Title/Abstract] OR "Autism Spectrum Disorder"[Title/Abstract] OR "Attention Deficit Hyperactivity Disorder"[Title/Abstract] OR "Pediatric Migraine"[Title/Abstract] OR "Landau-Kleffner Syndrome"[Title/Abstract] OR "West Syndrome"[Title/Abstract] OR "Dravet Syndrome"[Title/Abstract] OR "Lennox-Gastaut Syndrome"[Title/Abstract] OR "movement disorder*"[Title/Abstract] OR "myotonic disorder*"[Title/Abstract] OR "peripheral nerve disease*"[Title/Abstract]) OR ("Lung Diseases"[MeSH Terms] OR "asthma"[Title/Abstract] OR "Chronic Obstructive Pulmonary Disease"[Title/Abstract] OR "Pulmonary"[Title/Abstract] OR "Pulmonary Disease"[Title/Abstract] OR "Lung"[Title/Abstract] OR "Lung Disease"[Title/Abstract] OR "bronchitis"[Title/Abstract] OR "emphysema"[Title/Abstract] OR ("Disease"[All Fields] AND "Lung"[All Fields]) OR ("Diseases"[All Fields] AND "Lung"[All Fields]) OR "Lung Disease"[Title/Abstract] OR "Pulmonary Disease"[Title/Abstract] OR ("Disease"[All Fields] AND "Pulmonary"[All Fields]) OR ("Diseases"[All Fields] AND "Pulmonary"[All Fields]) OR "Pulmonary Diseases"[Title/Abstract] OR "Respiratory Tract Diseases"[Title/Abstract] OR "Acute Chest Syndrome"[Title/Abstract] OR "alpha 1-Antitrypsin Deficiency"[Title/Abstract] OR "Cystic Fibrosis"[Title/Abstract] OR "Hemoptysis"[Title/Abstract] OR "Hepatopulmonary Syndrome"[Title/Abstract] OR ("Hypertension"[All Fields] AND "Pulmonary"[All Fields]) OR "Familial Primary Pulmonary Hypertension"[Title/Abstract] OR "Persistent Fetal Circulation Syndrome"[Title/Abstract] OR "Pulmonary Arterial Hypertension"[Title/Abstract] OR "Lung Abscess"[Title/Abstract] OR "Blastomycosis"[Title/Abstract] OR ("Pneumonia"[All Fields] AND "Pneumocystis"[All Fields]) OR "Pulmonary Aspergillosis"[Title/Abstract] OR "Sarcoidosis"[Title/Abstract] OR "Pulmonary"[Title/Abstract] OR ("Pulmonary Disease"[All Fields] AND "Chronic Obstructive"[All Fields]) OR "Pneumonia"[Title/Abstract] OR "Respiratory Distress Syndrome"[Title/Abstract] OR "Scimitar Syndrome"[Title/Abstract] OR "Solitary Pulmonary Nodule"[Title/Abstract]) OR ("Gastrointestinal Diseases"[MeSH Terms] OR "gastroesophageal reflux"[Title/Abstract] OR "GERD"[Title/Abstract] OR "GORD"[Title/Abstract] OR "heartburn"[Title/Abstract] OR "Esophagitis"[Title/Abstract] OR "oesophagitis"[Title/Abstract] OR "liver disease"[Title/Abstract] OR "Hepatitis"[Title/Abstract] OR "cirrhosis"[Title/Abstract] OR "fibrosis"[Title/Abstract] OR "fatty liver"[Title/Abstract] OR "alcoholic liver disease"[Title/Abstract] OR "alcoholic hepatitis"[Title/Abstract] OR "Peptic Ulcer"[Title/Abstract] OR "duodenal ulcer"[Title/Abstract] OR "gastroduodenal ulcer"[Title/Abstract] OR "gallbladder disease"[Title/Abstract] OR "cholelithiasis"[Title/Abstract] OR "choledocholithiasis"[Title/Abstract] OR "cholangitis"[Title/Abstract] OR "biliary"[Title/Abstract] OR "irritable bowel syndrome"[Title/Abstract] OR "irritable colon"[Title/Abstract] OR "dysphagia"[Title/Abstract] OR ("Disease"[All Fields] AND "Gastrointestinal"[All Fields]) OR ("Diseases"[All Fields] AND "Gastrointestinal"[All Fields]) OR "Gastrointestinal Disease"[Title/Abstract] OR "Gastrointestinal Disorders"[Title/Abstract] OR "Gastrointestinal Disorder"[Title/Abstract] OR "Functional Gastrointestinal Disorders"[Title/Abstract] OR "Functional Gastrointestinal Disorder"[Title/Abstract] OR ("Gastrointestinal Disorder"[All Fields] AND "Functional"[All Fields]) OR ("Gastrointestinal Disorders"[All Fields] AND "Functional"[All Fields]) OR "Cholera Infantum"[Title/Abstract] OR "Esophageal Diseases"[Title/Abstract] OR "Barrett Esophagus"[Title/Abstract] OR "Deglutition Disorders"[Title/Abstract] OR (("diverticulum"[MeSH Terms] OR "diverticulum"[All Fields] OR "diverticulosis"[All Fields]) AND "Esophageal"[All Fields]) OR "Gastric Varices"[Title/Abstract] OR "Esophageal Atresia"[Title/Abstract] OR "Esophageal Cyst"[Title/Abstract] OR "Esophageal Fistula"[Title/Abstract] OR "Esophageal Neoplasms"[Title/Abstract] OR "Esophageal Perforation"[Title/Abstract] OR "Esophageal Stenosis"[Title/Abstract] OR "Esophagitis"[Title/Abstract] OR "Appendicitis"[Title/Abstract] OR "Cholera Morbus"[Title/Abstract] OR "Diverticular Diseases"[Title/Abstract] OR "Dysentery"[Title/Abstract] OR "Enteritis"[Title/Abstract] OR "Enterocolitis"[Title/Abstract] OR "Gastritis"[Title/Abstract] OR "Mucositis"[Title/Abstract] OR "Proctitis"[Title/Abstract] OR "Inflammatory Bowel Diseases"[Title/Abstract] OR "Hematemesis"[Title/Abstract] OR "Melena"[Title/Abstract] OR "Peptic Ulcer Hemorrhage"[Title/Abstract] OR "Esophageal Neoplasms"[Title/Abstract] OR "Gastrointestinal Stromal Tumors"[Title/Abstract] OR "Intestinal Neoplasms"[Title/Abstract] OR "Stomach Neoplasms"[Title/Abstract] OR "Zollinger-Ellison Syndrome"[Title/Abstract] OR "Cecal Diseases"[Title/Abstract] OR "Colonic Diseases"[Title/Abstract] OR "Duodenal Diseases"[Title/Abstract] OR "Dysentery"[Title/Abstract] OR "Enteritis"[Title/Abstract] OR "Enterocolitis"[Title/Abstract] OR "HIV Enteropathy"[Title/Abstract] OR "Ileal Diseases"[Title/Abstract] OR "Intestinal Atresia"[Title/Abstract] OR ("Intestinal Diseases"[All Fields] AND "Parasitic"[All Fields]) OR "Intestinal Fistula"[Title/Abstract] OR "Intestinal Neoplasms"[Title/Abstract] OR "Intestinal Obstruction"[Title/Abstract] OR "Intestinal Perforation"[Title/Abstract] OR "Intestinal Polyposis"[Title/Abstract] OR "Jejunal Diseases"[Title/Abstract] OR "Malabsorption Syndromes"[Title/Abstract] OR "Mesenteric Ischemia"[Title/Abstract] OR "Mesenteric Vascular Occlusion"[Title/Abstract] OR "Pneumatosis Cystoides Intestinalis"[Title/Abstract] OR "Protein-Losing Enteropathies"[Title/Abstract] OR "Rectal Diseases"[Title/Abstract] OR "Rumination Syndrome"[Title/Abstract] OR "Achlorhydria"[Title/Abstract] OR (("diverticulum"[MeSH Terms] OR "diverticulum"[All Fields] OR "diverticulosis"[All Fields]) AND "Stomach"[All Fields]) OR "Duodenogastric Reflux"[Title/Abstract] OR "Gastric Antral Vascular Ectasia"[Title/Abstract] OR "Gastric Dilatation"[Title/Abstract] OR "Gastric Outlet Obstruction"[Title/Abstract] OR "Gastritis"[Title/Abstract] OR "Gastroparesis"[Title/Abstract] OR "Peptic Ulcer"[Title/Abstract] OR "Postgastrectomy Syndromes"[Title/Abstract] OR "Stomach Neoplasms"[Title/Abstract] OR "Stomach Rupture"[Title/Abstract] OR "Stomach Volvulus"[Title/Abstract] OR "Zollinger-Ellison Syndrome"[Title/Abstract] OR "Visceral Prolapse"[Title/Abstract] OR "Reflux"[Title/Abstract] OR "Appendicitis"[Title/Abstract] OR "Barrett's Esophagus"[Title/Abstract] OR "Celiac Disease"[Title/Abstract] OR "Celiac"[Title/Abstract] OR "Constipation"[Title/Abstract] OR "Crohn's Disease"[Title/Abstract] OR "Dumping Syndrome"[Title/Abstract] OR "Gallstones"[Title/Abstract] OR "Indigestion"[Title/Abstract] OR "Dyspepsia"[Title/Abstract] OR

"Hernia"[Title/Abstract] OR "Intestinal Pseudo-obstruction"[Title/Abstract] OR "obstruction"[Title/Abstract] OR "Menetrier's Disease"[Title/Abstract] OR "Peptic Ulcers"[Title/Abstract] OR "ulcer"[Title/Abstract] OR "Short Bowel Syndrome"[Title/Abstract] OR "Ulcerative Colitis"[Title/Abstract]) OR (("Neoplasms"[MeSH Terms] OR "Cancer"[Title/Abstract] OR "carcinoma"[Title/Abstract] OR "neoplasia"[Title/Abstract] OR "tumor"[Title/Abstract] OR "neoplasm"[Title/Abstract] OR "maligna*"[Title/Abstract]) AND "German"[Language] AND "English"[Language] AND "French"[Language]) OR ("hypothyroidism"[Title/Abstract] OR "myxedema"[Title/Abstract] OR "hyperthyroidism"[Title/Abstract] OR "thyrotoxicosis"[Title/Abstract] OR "Graves disease"[Title/Abstract] OR "thyroiditis"[Title/Abstract] OR "Hashimoto's"[Title/Abstract] OR "Rheumatoid Arthritis"[Title/Abstract] OR "Felty syndrome"[Title/Abstract] OR "Rheumatoid nodule"[Title/Abstract] OR "Rheumatoid vasculitis"[Title/Abstract] OR "adult onset Still's disease"[Title/Abstract] OR "dermatomyositis"[Title/Abstract] OR "polymyositis"[Title/Abstract] OR "idiopathic inflammatory myopathies"[Title/Abstract] OR "Myositis"[Title/Abstract] OR "ankylosing spondylitis"[Title/Abstract] OR "spondylitis ankylosing"[Title/Abstract] OR "Rheumatoid spondylitis"[Title/Abstract] OR "vertebral ankylosis"[Title/Abstract] OR "spondyloarthropathy"[Title/Abstract] OR "bechterew*"[Title/Abstract] OR "Bechterew's disease"[Title/Abstract] OR "marie strumpell*"[Title/Abstract] OR "Inflammatory bowel disease"[Title/Abstract] OR "Crohn's"[Title/Abstract] OR "Ulcerative Colitis"[Title/Abstract] OR "Uveitis"[Title/Abstract] OR "uveiti*"[Title/Abstract] OR "panuveitis"[Title/Abstract] OR "iridocyclitis"[Title/Abstract] OR "iridocycliti*"[Title/Abstract] OR "anterior scleritis"[Title/Abstract] OR "iritis"[Title/Abstract] OR "iriti*"[Title/Abstract] OR "pars planitis"[Title/Abstract] OR "Lupus"[Title/Abstract] OR "Lupus erythematosus"[Title/Abstract] OR "Systemic lupus erythematosus"[Title/Abstract] OR "Lupus vulgaris"[Title/Abstract] OR "Psoriasis"[Title/Abstract] OR "palmoplantaris pustulosis"[Title/Abstract] OR "pustular psoriasis"[Title/Abstract] OR "Pemphigus"[Title/Abstract] OR "pemphigus vulgaris"[Title/Abstract] OR "pemphigus foliaceus"[Title/Abstract] OR "primary biliary cirrhosis"[Title/Abstract] OR "liver cirrhosis"[Title/Abstract] OR "Bullous Pemphigoid"[Title/Abstract] OR "pemphigoid"[Title/Abstract] OR "myasthenia"[Title/Abstract] OR "Myasthenia Gravis"[Title/Abstract] OR "anti-acetylcholine receptor antibody"[Title/Abstract] OR "anti-muscle specific kinase antibody"[Title/Abstract] OR "seronegative MG"[Title/Abstract] OR "Myasthenia Gravis"[Title/Abstract] OR "myasthenia"[Title/Abstract] OR "congenital myasthenic syndrome"[Title/Abstract] OR "systemic sclerosis"[Title/Abstract] OR "scleroderma"[Title/Abstract] OR "pernicious anaemia"[Title/Abstract] OR "atrophic gastritis"[Title/Abstract] OR "type A gastritis"[Title/Abstract] OR "macrocytic anaemia"[Title/Abstract] OR "cobalamin deficiency"[Title/Abstract] OR "vitamin B12 deficiency"[Title/Abstract] OR "Gastritis"[Title/Abstract] OR "idiopathic thrombocytopenic purpura"[Title/Abstract] OR "thrombocytopeni*"[Title/Abstract] OR "Addison's disease"[Title/Abstract] OR "Addison disease"[Title/Abstract] OR "primary adrenal insufficiency"[Title/Abstract] OR "primary adrenocortical insufficiency"[Title/Abstract] OR "adrenal-Cortex-Diseases"[Title/Abstract] OR "adrenal-Gland-Diseases"[Title/Abstract] OR "adrenal-Insufficiency"[Title/Abstract] OR "Wegener Granulomatosis"[Title/Abstract] OR "vasculitis"[Title/Abstract] OR "wegener vascul*"[Title/Abstract] OR "small vessel vascul*"[Title/Abstract] OR "Celiac"[Title/Abstract] OR "sprue"[Title/Abstract] OR "gluten sensitive enteropathy"[Title/Abstract] OR "villous atrophy"[Title/Abstract] OR "antigliadin"[Title/Abstract] OR "endomyseal"[Title/Abstract] OR "tissue transglutaminase"[Title/Abstract] OR "Alopecia areata"[Title/Abstract] OR "Alopecia totalis"[Title/Abstract] OR "Alopecia universalis"[Title/Abstract] OR "Ophiasis"[Title/Abstract] OR "non-scarring hair loss"[Title/Abstract] OR "Vitiligo"[Title/Abstract] OR "leukoderma"[Title/Abstract] OR "leucoderma"[Title/Abstract] OR "Sjogren's syndrome"[Title/Abstract]) OR ("gout"[Title/Abstract] OR "gouty arthritis"[Title/Abstract] OR "Arthritis"[Title/Abstract] OR "Osteoarthritis"[Title/Abstract] OR "hip osteoarthritis"[Title/Abstract] OR "knee osteoarthritis"[Title/Abstract] OR "spine osteoarthritis"[Title/Abstract] OR "fibromyalgia"[Title/Abstract] OR "fibromyositis"[Title/Abstract] OR "fibrositis"[Title/Abstract] OR "bone disease*"[Title/Abstract] OR "coxa magna"[Title/Abstract] OR "coxa valga"[Title/Abstract] OR "Osteitis"[Title/Abstract] OR "Osteochondritis"[Title/Abstract] OR "Osteochondrosis"[Title/Abstract] OR "Osteonecrosis"[Title/Abstract] OR "spinal disease*"[Title/Abstract] OR "cartilage disease*"[Title/Abstract] OR "Fasciitis"[Title/Abstract] OR "foot deformities"[Title/Abstract] OR "foot deformity"[Title/Abstract] OR "hand deformities"[Title/Abstract] OR "hand deformity"[Title/Abstract] OR "jaw disease*"[Title/Abstract] OR "mandibular disease*"[Title/Abstract] OR "maxillary disease*"[Title/Abstract] OR "Ankylosis"[Title/Abstract] OR "Arthralgia"[Title/Abstract] OR "Arthritis"[Title/Abstract] OR "Arthrogryposis"[Title/Abstract] OR "Bursitis"[Title/Abstract] OR "Contracture"[Title/Abstract] OR "femoracetabular impingement"[Title/Abstract] OR "hallux limitus"[Title/Abstract] OR "hallux rigidus"[Title/Abstract] OR "Hemarthrosis"[Title/Abstract] OR "Hydrarthrosis"[Title/Abstract] OR "Osteoarthropathy"[Title/Abstract] OR "Synovitis"[Title/Abstract] OR "Arthrogryposis"[Title/Abstract] OR "Myositis"[Title/Abstract] OR "myotonic disorders"[Title/Abstract] OR "myotonic disorder"[Title/Abstract] OR "Myotoxicity"[Title/Abstract] OR "Arthrogryposis"[Title/Abstract] OR "campomelic dysplasia"[Title/Abstract] OR "Synostosis"[Title/Abstract] OR "pectus carinatum"[Title/Abstract] OR "Gastroschisis"[Title/Abstract] OR "craniofacial abnormalities"[Title/Abstract] OR "Arthritis"[Title/Abstract] OR "Juvenile Arthritis"[Title/Abstract] OR "Rheumatoid Arthritis"[Title/Abstract] OR "Sternocostoclavicular Hyperostosis"[Title/Abstract] OR "Osteoarthritis"[Title/Abstract] OR "polymyalgia rheumatica"[Title/Abstract] OR "rheumatic fever"[Title/Abstract]) OR ("Hematologic Diseases"[MeSH Terms] OR "Anemia"[Title/Abstract] OR "Hemoglobinopathies"[Title/Abstract] OR "Thalassemia"[Title/Abstract] OR "Sickle Cell Disease"[Title/Abstract] OR "Hemophilia"[Title/Abstract] OR "Leukemia"[Title/Abstract] OR "Lymphoma"[Title/Abstract] OR "Myelodysplastic Syndromes"[Title/Abstract] OR "Myeloproliferative Disorders"[Title/Abstract] OR "Platelet Disorders"[Title/Abstract] OR "Hemostatic Disorders"[Title/Abstract] OR "Coagulation Disorders"[Title/Abstract] OR "Bleeding Disorders"[Title/Abstract] OR "Blood Coagulation Disorders"[Title/Abstract] OR "Iron Overload"[Title/Abstract] OR "Hemochromatosis"[Title/Abstract] OR "Polycythemia Vera"[Title/Abstract] OR "Leukopenia"[Title/Abstract] OR "Thrombocytopenia"[Title/Abstract] OR "Thrombosis"[Title/Abstract] OR "Hemostasis Disorders"[Title/Abstract] OR "Blood Transfusion"[Title/Abstract] OR "Bone Marrow Diseases"[Title/Abstract] OR "Aplastic Anemia"[Title/Abstract] OR "Lymphadenopathy"[Title/Abstract] OR "Multiple Myeloma"[Title/Abstract] OR "Blood Platelet Disorders"[Title/Abstract] OR "Blood Protein Disorders"[Title/Abstract] OR "Hematopoietic Stem Cell Transplantation"[Title/Abstract]) OR ("Skin Diseases"[MeSH Terms] OR "Dermatitis"[Title/Abstract] OR "Eczema"[Title/Abstract] OR "Psoriasis"[Title/Abstract] OR "Acne"[Title/Abstract] OR "Skin Neoplasms"[Title/Abstract] OR "Melanoma"[Title/Abstract] OR "Basal Cell Carcinoma"[Title/Abstract] OR "Squamous Cell Carcinoma"[Title/Abstract] OR "Urticaria"[Title/Abstract] OR "Hives"[Title/Abstract] OR "Alopecia"[Title/Abstract] OR "Hair Loss"[Title/Abstract] OR "Vitiligo"[Title/Abstract] OR "Rosacea"[Title/Abstract] OR "Impetigo"[Title/Abstract] OR "Fungal Skin Infections"[Title/Abstract] OR "Tinea"[Title/Abstract] OR "Scabies"[Title/Abstract] OR "Lichen Planus"[Title/Abstract] OR "Bullous Pemphigoid"[Title/Abstract] OR "Pemphigus"[Title/Abstract] OR "Herpes Zoster"[Title/Abstract] OR "Shingles"[Title/Abstract] OR "Leprosy"[Title/Abstract] OR "Hansen's Disease"[Title/Abstract] OR "Skin Ulcer"[Title/Abstract] OR "Decubitus Ulcer"[Title/Abstract] OR "Cellulitis"[Title/Abstract] OR "Erythema"[Title/Abstract] OR "Rash"[Title/Abstract] OR "Dermatologic Agents"[Title/Abstract] OR "Skin Care"[Title/Abstract] OR "Skin Aging"[Title/Abstract] OR "Wound Healing"[Title/Abstract] OR "Burns"[Title/Abstract] OR "Frostbite"[Title/Abstract] OR "Pressure Ulcer"[Title/Abstract] OR "Sunburn"[Title/Abstract] OR "Pigmentation Disorders"[Title/Abstract] OR "Melasma"[Title/Abstract] OR "Hyperpigmentation"[Title/Abstract] OR "Hypopigmentation"[Title/Abstract]) OR ("Urologic

Diseases"[MeSH Terms] OR "genital diseases, male"[MeSH Terms] OR "genital diseases, female"[MeSH Terms] OR "Kidney Diseases"[Title/Abstract] OR "Urinary Tract Infections"[Title/Abstract] OR "UTI"[Title/Abstract] OR "Bladder Diseases"[Title/Abstract] OR "Cystitis"[Title/Abstract] OR "Urethritis"[Title/Abstract] OR "Prostatitis"[Title/Abstract] OR "Erectile Dysfunction"[Title/Abstract] OR "Prostate Cancer"[Title/Abstract] OR "Bladder Cancer"[Title/Abstract] OR "Kidney Stones"[Title/Abstract] OR "Nephrolithiasis"[Title/Abstract] OR "Urinary Incontinence"[Title/Abstract] OR "Overactive Bladder"[Title/Abstract] OR "Interstitial Cystitis"[Title/Abstract] OR "Pelvic Organ Prolapse"[Title/Abstract] OR "Endometriosis"[Title/Abstract] OR "Polycystic Ovary Syndrome"[Title/Abstract] OR "PCOS"[Title/Abstract] OR "Uterine Fibroids"[Title/Abstract] OR "Menstrual Disorders"[Title/Abstract] OR "Ovarian Cysts"[Title/Abstract] OR "Gynecologic Cancer"[Title/Abstract] OR "Cervical Cancer"[Title/Abstract] OR "Ovarian Cancer"[Title/Abstract] OR "Endometrial Cancer"[Title/Abstract] OR "Pelvic Inflammatory Disease"[Title/Abstract] OR "PID"[Title/Abstract] OR "Sexually Transmitted Diseases"[Title/Abstract] OR "HIV/AIDS"[Title/Abstract] OR "Genital Herpes"[Title/Abstract] OR "Gonorrhea"[Title/Abstract] OR "Chlamydia"[Title/Abstract] OR "Syphilis"[Title/Abstract] OR "Human Papillomavirus"[Title/Abstract] OR "Urological Agents"[Title/Abstract] OR "Renal Failure"[Title/Abstract] OR "Chronic Kidney Disease"[Title/Abstract] OR "Acute Kidney Injury"[Title/Abstract]) OR ("Communicable Diseases"[MeSH Terms] OR "Parasitic Diseases"[MeSH Terms] OR "Bacterial Infections"[Title/Abstract] OR "Viral Infections"[Title/Abstract] OR "Fungal Infections"[Title/Abstract] OR "Protozoan Infections"[Title/Abstract] OR "Helminthiasis"[Title/Abstract] OR "Worm Infections"[Title/Abstract] OR "Malaria"[Title/Abstract] OR "Tuberculosis"[Title/Abstract] OR "HIV"[Title/Abstract] OR "AIDS"[Title/Abstract] OR "Hepatitis"[Title/Abstract] OR "Influenza"[Title/Abstract] OR "COVID-19"[Title/Abstract] OR "SARS-CoV-2"[Title/Abstract] OR "Zika Virus"[Title/Abstract] OR "Dengue"[Title/Abstract] OR "Chikungunya"[Title/Abstract] OR "Lyme Disease"[Title/Abstract] OR "Syphilis"[Title/Abstract] OR "Gonorrhea"[Title/Abstract] OR "Chlamydia"[Title/Abstract] OR "Herpes Simplex"[Title/Abstract] OR "Herpes Zoster"[Title/Abstract] OR "Candidiasis"[Title/Abstract] OR "Aspergillosis"[Title/Abstract] OR "Cryptococcosis"[Title/Abstract] OR "Schistosomiasis"[Title/Abstract] OR "Leishmaniasis"[Title/Abstract] OR "Toxoplasmosis"[Title/Abstract] OR "Trichomoniasis"[Title/Abstract] OR "Giardiasis"[Title/Abstract] OR "Amoebiasis"[Title/Abstract] OR "Hookworm Infection"[Title/Abstract] OR "Ascariasis"[Title/Abstract] OR "Filariasis"[Title/Abstract] OR "Onchocerciasis"[Title/Abstract] OR "Trachoma"[Title/Abstract] OR "Antimicrobial Resistance"[Title/Abstract] OR "Antibiotic Resistance"[Title/Abstract]) OR ("Cataract"[Title/Abstract] OR "Glaucoma"[Title/Abstract] OR "Macular Degeneration"[Title/Abstract] OR "Diabetic Retinopathy"[Title/Abstract] OR "Uveitis"[Title/Abstract] OR "Conjunctivitis"[Title/Abstract] OR "Pink Eye"[Title/Abstract] OR "Corneal Diseases"[Title/Abstract] OR "Keratitis"[Title/Abstract] OR "Dry Eye Syndromes"[Title/Abstract] OR "Strabismus"[Title/Abstract] OR "Crossed Eyes"[Title/Abstract] OR "Amblyopia"[Title/Abstract] OR "Lazy Eye"[Title/Abstract] OR "Blepharitis"[Title/Abstract] OR "Color Vision Defects"[Title/Abstract] OR "Night Blindness"[Title/Abstract] OR "Refractive Errors"[Title/Abstract] OR "Myopia"[Title/Abstract] OR "Hyperopia"[Title/Abstract] OR "Astigmatism"[Title/Abstract] OR "Presbyopia"[Title/Abstract] OR "Eye Infections"[Title/Abstract] OR "Ocular Hypertension"[Title/Abstract] OR "Retinopathy of Prematurity"[Title/Abstract] OR "Vitreous Detachment"[Title/Abstract] OR "Retinal Detachment"[Title/Abstract])) AND ("Multiple Sclerosis"[MeSH Terms] OR "Multiple Sclerosis"[Title/Abstract]) AND ("Pediatrics"[MeSH Terms] OR "Child"[MeSH Terms] OR "Adolescent"[MeSH Terms] OR "child, preschool"[MeSH Terms] OR "pediatric"[Title/Abstract] OR "Child"[Title/Abstract] OR "Adolescent"[Title/Abstract])) NOT "Case Reports"[Publication Type]) NOT "Editorial"[Publication Type]) NOT "Letter"[Publication Type]) NOT "News"[Publication Type]) AND "Humans"[MeSH Terms] AND ("English"[Language] OR "German"[Language] OR "French"[Language])

Supplementary Table S3: Search strategy for Embase (produced by Elsevier)		
No.	Domain	Search terms in Embase
1	Disease	'Multiple sclerosis'/exp
2	Population	('pediatrics'/exp OR 'child'/exp OR 'adolescent'/exp)
3	Comorbidities	('comorbidity'/exp OR 'multiple chronic conditions'/exp)
	Combined search	'Multiple sclerosis'/exp AND ('pediatrics'/exp OR 'child'/exp OR 'adolescent'/exp) AND ('comorbidity'/exp OR 'multiple chronic conditions'/exp)
